# Allogeneic haematopoietic cell transplantation promotes atherosclerosis in mice via CD8^+^ T cells

**DOI:** 10.1093/cvr/cvaf229

**Published:** 2025-11-18

**Authors:** Ivana Jorgacevic, Haroon Shaikh, Hla Ali, Maja Bundalo, Sarah Schäfer, Michael A G Kern, Maike Büttner-Herold, Simone Reu-Hofer, Clément Cochain, Hendrik Bartolomaeus, Antoine-Emmanuel Saliba, Melanie Rösch, Giuseppe Rizzo, Estibaliz Arellano Viera, Juan Gamboa Vargas, Friederike Berberich-Siebelt, Wolfgang Herr, Louis Boon, Andreas Rosenwald, Elke Butt, Heike M Hermanns, Andreas Beilhack, Alma Zernecke

**Affiliations:** Institute of Experimental Biomedicine, University Hospital Würzburg, Josef-Schneider-Str. 2, D16, Würzburg 97080, Germany; Department of Internal Medicine II, University Hospital Würzburg, Würzburg, Germany; Institute of Experimental Biomedicine, University Hospital Würzburg, Josef-Schneider-Str. 2, D16, Würzburg 97080, Germany; Institute of Experimental Biomedicine, University Hospital Würzburg, Josef-Schneider-Str. 2, D16, Würzburg 97080, Germany; Institute of Experimental Biomedicine, University Hospital Würzburg, Josef-Schneider-Str. 2, D16, Würzburg 97080, Germany; Department of Internal Medicine II, University Hospital Würzburg, Würzburg, Germany; Department of Nephropathology, Institute of Pathology, Friedrich-Alexander-University Erlangen-Nuremberg (FAU) and University Hospital, Erlangen, Germany; Institute of Pathology, Julius-Maximilians-University Würzburg, Würzburg, Germany; Institute of Experimental Biomedicine, University Hospital Würzburg, Josef-Schneider-Str. 2, D16, Würzburg 97080, Germany; INSERM U970, Paris Cardiovascular Research Center, Université Paris Cité, Paris, France; Institute of Experimental Biomedicine, University Hospital Würzburg, Josef-Schneider-Str. 2, D16, Würzburg 97080, Germany; Helmholtz-Center for Infection Research (HZI), Helmholtz Institute for RNA-based Infection Research (HIRI), Würzburg, Germany; Institute of Experimental Biomedicine, University Hospital Würzburg, Josef-Schneider-Str. 2, D16, Würzburg 97080, Germany; Institute of Experimental Biomedicine, University Hospital Würzburg, Josef-Schneider-Str. 2, D16, Würzburg 97080, Germany; Department of Internal Medicine II, University Hospital Würzburg, Würzburg, Germany; Department of Internal Medicine II, University Hospital Würzburg, Würzburg, Germany; Institute of Pathology, Julius-Maximilians-University Würzburg, Würzburg, Germany; Department of Internal Medicine III, University Hospital Regensburg, Regensburg, Germany; JJP Biologics, Bobrowiecka 6, Warsaw 00-728, Poland; Institute of Pathology, Julius-Maximilians-University Würzburg, Würzburg, Germany; Comprehensive Cancer Centre Mainfranken, Würzburg University Hospital, Würzburg, Germany; Institute of Experimental Biomedicine, University Hospital Würzburg, Josef-Schneider-Str. 2, D16, Würzburg 97080, Germany; Department of Internal Medicine II, University Hospital Würzburg, Würzburg, Germany; Department of Internal Medicine II, University Hospital Würzburg, Würzburg, Germany; Institute of Experimental Biomedicine, University Hospital Würzburg, Josef-Schneider-Str. 2, D16, Würzburg 97080, Germany

**Keywords:** Atherosclerosis, Haematopoietic cell transplantation, T cells, Graft-versus-host disease

## Abstract

**Aims:**

Patients undergoing allogeneic haematopoietic cell transplantation (allo-HCT) are at a risk of developing graft-vs.-host disease (GvHD) and are afflicted with an increased incidence of cardiovascular events. Whether allo-HCT contributes to atherosclerosis progression has not been addressed experimentally.

**Methods and results:**

Here, we applied a novel minor histocompatibility antigen mismatch allo-HCT mouse model by transplanting C57BL/6 mice deficient for the LDL receptor (B6.*Ldlr*^−/−^) with bone marrow (BM) or BM and T cells (BMT) from BALB/b donors (BALB/b, H-2^b^ → B6.*Ldlr*^−/−^, and H-2^b^), and feeding recipients a western diet. Mild clinical GvHD symptoms ensued with low disease activity in typical GvHD target organs. However, allogeneic BMT recipients developed increased atherosclerotic lesions compared with mice receiving BM only or syngeneic BMT recipients. Atherosclerotic lesions showed a heightened infiltration of effector CD8^+^ T cells in the aorta of BMT recipients. Furthermore, BMT recipients exhibited significantly higher serum cholesterol levels than BM-recipients. Notably, CD8^+^ T-cell depletion in B6.*Ldlr*^−/−^ BMT recipients reduced atherosclerotic lesion formation and decreased cholesterol levels.

**Conclusion:**

These data may provide a novel mechanistic underpinning for the clinically observed increased incidence of cardiovascular disease in long-term allo-HCT survivors. Moreover, we have identified CD8^+^ T cells as potential targets for mitigating GvHD-induced atherosclerosis.

## Graphical Abstract

**Graphical Abstract:**
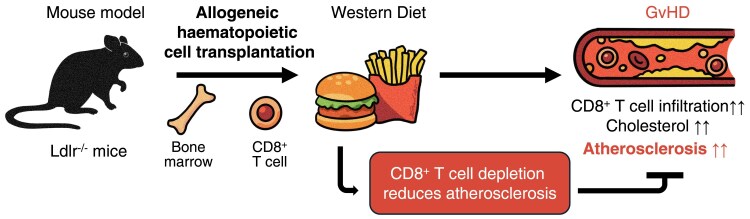

## 1. Introduction

Allogeneic haematopoietic cell transplantation (allo-HCT) represents the main and often only treatment option for many malignant and non-malignant haematological disorders. Although allo-HCT protocols have been continuously improved over the last decades and patients’ life expectancy has increased, post allo-HCT patients are at a risk of developing complications, such as life-threatening graft-vs.-host disease (GvHD), but also pulmonary disease, endocrine dysfunction, infertility, or cataracts.^1–4^ A particular concern is the increased cumulative incidence of cardiovascular events in long-term survivors^1,2,5^ and the more than two-fold increased risk of premature cardiovascular death compared with the general population.^1,6,7^

GvHD, as the primary adverse side effect of allo-HCT, remains the leading cause of morbidity and mortality after allo-HCT.^6,8^ GvHD is furthermore recognized as a risk factor for the onset of dyslipidaemia, hypertension, and diabetes mellitus,^9–11^ all of which contribute to cardiovascular risk.^12–14^ However, whether GvHD directly affects survival and cardiovascular risk in allo-HCT patients remains controversial. While some studies show no differences between recipients of autologous and allo-HCT,^7^ others identify a clear association with active chronic GvHD.^15^

Cardiovascular disease and its manifestations, including stroke, myocardial infarction, and peripheral arterial disease, are caused by atherosclerosis, a chronic inflammatory disease affecting the arterial wall. Local and systemic inflammatory responses and immune cells drive atherosclerosis in the context of hyperlipidaemia. During atherosclerosis, intimal macrophages take up oxidized LDL (oxLDL), and other lipids deposited in the intima, leading to the formation of foam cells. Additionally, various subsets of macrophages, dendritic cells, and T cells accumulate within atherosclerotic lesions.^16–18^ Moreover, medial vascular smooth muscle cells migrate into the intima to form the fibrous cap, which, along with collagen, stabilizes the atherosclerotic lesion.^19,20^ As the plaque enlarges, a necrotic core forms from dying plaque cells. CD8^+^ T cells regulate early atherosclerotic lesion development and promote apoptosis of various plaque cell types, including smooth muscle cells, thereby contributing to necrotic core formation.^21,22^

In GvHD, donor T cells in the haematopoietic cell graft recognize recipient tissue antigens, which trigger an allo-reactive T-cell response. While treatment with anti-thymocyte globulin, post-transplant cyclophosphamide, T-cell depletion, and costimulatory blockade have mitigated GvHD severity, treatment with these agents is associated with an increased risk of relapse or infections.^23^ Activation of donor T cells by recipient haematopoietic and non-haematopoietic antigen-presenting cells (APC) enables donor T cells to acquire cytolytic capacity, leading to the attack of recipient cells.^8^ After a brief priming phase of allo-reactive T cells in secondary lymphoid organs,^24,25^ acute GvHD manifests in target organs, such as the gastrointestinal tract, liver, and skin.^8,25–27^ Furthermore, endothelial damage and dysfunction have been observed in experimental models and in patients undergoing allo-HCT,^28^ resulting in transplantation-associated-microangiopathy,^29^ veno-occlusive disease,^30^ capillary leak syndrome,^31^ and diffuse alveolar haemorrhage.^32^ While donor T cells and their effector mechanisms have also been linked to atherosclerotic lesion formation,^21–23^ the direct relationship between allo-HCT and atherosclerosis had not been studied experimentally.

Here, we investigated whether allo-HCT contributes to atherosclerosis by establishing a novel mouse model using a minor histocompatibility antigen (miHAg) mismatch transplant approach in atherosclerosis-prone LDL receptor-deficient (*Ldlr*^−/−^) mice. Our findings demonstrate that transplanting atherosclerosis-prone C57BL/6 *Ldlr*^−/−^ mice with allogeneic BALB/b donor BM and T cells (BMT) accelerates atherosclerotic lesion formation and elevates serum cholesterol levels, and that these effects are at least partly driven by donor CD8^+^ T cells.

## 2. Methods

Please see Supplementary material online for additional information.

### 2.1 Mice and allo-HCT

C57BL/6.*Ldlr*^−/−^ (B6.*Ldlr*^−/−^) recipients were myeloablatively irradiated with 9 Gy and intravenously injected with 5 × 10^6^ wild-type (WT) BALB/b donor bone marrow (BM) with/without 5 × 10^6^ WT splenic T cells. The occurrence of clinical GvHD symptoms (posture, activity, fur, skin, and weight loss)^33^ was monitored daily. To induce atherosclerosis, recipient mice were placed on a western diet (WD; 21% fat and 0.15% cholesterol) that started 10 days after the allo-HCT. A group of mice in addition received once weekly intraperitoneal injections of anti-mouse-CD8β IgG2b antibody (Ab) (clone YTS156.7.7, referred to as anti-CD8β) or isotype-matched irrelevant rat-anti-Phyt1 (clone AFRC-MAC51, referred to as isotype). Animals were euthanized by an overdose of isoflurane anaesthesia (5% concentration), cervical dislocation, and subsequent exsanguination and organ isolation. All animal studies conformed to the Directive 2010/63/EU of the European Parliament and had been approved by local authorities (Regierung von Unterfranken, Würzburg, Germany).

### 2.2 Atherosclerotic lesion quantification and immunohistochemistry

Arteries were flushed and perfusion-fixed *in situ* using 4% buffered paraformaldehyde (PFA) in phosphate buffered saline. Hearts were snap-frozen and cut into 5 µm transverse sections and stained with aldehyde-fuchsin solution for plaque size quantification. Quantification of lesion size in the aorta was assessed after staining lipid depositions using Oil-Red-O. Necrotic core formation was quantified in sections stained with haematoxylin and eosin.^34^ Macrophages and SMC were detected by immunofluorescence staining using anti-Mac-2 and anti-αSMA-Cy3 antibody. Plaque size and cell content were quantified by computerized image analysis and investigators blinded to the group distributions.^34^ The plaque vulnerability index was calculated by the ratio of monocyte/macrophage content to the sum of SMC and collagen area.

### 2.3 Histological analysis

Liver, skin, colon, and small intestine were fixed in 4% PFA before embedding in paraffin. Five micrometre haematoxylin and eosin-stained sections were scored by experienced pathologists blinded to experimental history. Briefly, for the assessment of intestinal probes inflammation, crypt apoptotic body counts and crypt lost/destruction were semi-quantitatively scored, and giant cells, ulceration/erosion, and architectural distortion were evaluated as present or absent. From the respective values a cumulative sum score was generated for each probe. For the analyses of skin probes, dermal infiltrates were semi-quantitatively scored, and acanthosis, spongiosis, hypergranulosis, lengthened/broadened rete ridges, basal vacuolization, sub-epidermal clefting, inflammatory cell exocytosis, apoptotic bodies, and scab were evaluated as present or absent in 10 separate high-power fields, and a sum score was generated from the mean values. For the assessment of liver tissue portal and lobular inflammation, stellate cell activation as well as liver cell regeneration was scored semi-quantitatively.

### 2.4 Flow cytometry

The aorta was cleaned of fat and enzymatically digested.^34^ Single-cell suspensions from blood, BM, spleen, aorta, and lymph nodes (LN) were stained with antibodies, and samples acquired with a FACS Celesta and analysed with FlowJo 10.0 software (BD Biosciences).

### 2.5 scRNA-sequencing

Single-cell RNA sequencing (scRNA-seq) of aortic CD45^+^ leucocytes was performed, as described.^35^ Briefly, aortas were collected, minced, and digested in Roswell Park Memorial Institute (RPMI) medium containing collagenase I and XI, hyaluronidase, and DNase. Cell suspensions were labelled with anti-CD45.2-Alexa Fluor 488, fixable viability dye e780, and anti-mouse TotalSeqA-Hashtag antibodies. Viable CD45^+^ cells were sorted using a FACS Aria III (BD Biosciences), counted, and loaded into the 10x Genomics Chromium. Libraries were generated with the Chromium Single-Cell 3′ Reagents Kit v3. Sequencing was performed with S1 or S2 100 bp flowcell with Novaseq 6000 platform (Illumina). 10x Genomics data were demultiplexed using Cell Ranger software (version 3.1.0). Mouse GRCm38-mm10 reference genome was used for the alignment and counting steps. The gene-barcode matrix obtained from Cell Ranger was further analysed using Seurat package from R (www.satijalab.org, version 4.1.1). Single-cell RNA sequencing data have been deposited in Gene Expression Omnibus (GEO) with accession ID GSE291588.

### 2.6 Statistical analysis

Data are presented as mean ± standard deviation (std dev). Comparisons were performed via unpaired non-parametric Mann–Whitney *U* test with Prism software (GraphPad Prism 10). Differences in animal survival (Kaplan–Meier survival curves) were analysed by log-rank test. Differences were deemed statistically significant when *P* < 0.05.

## 3. Results

### 3.1 Subclinical GvHD aggravates atherosclerosis

**Figure 1:**
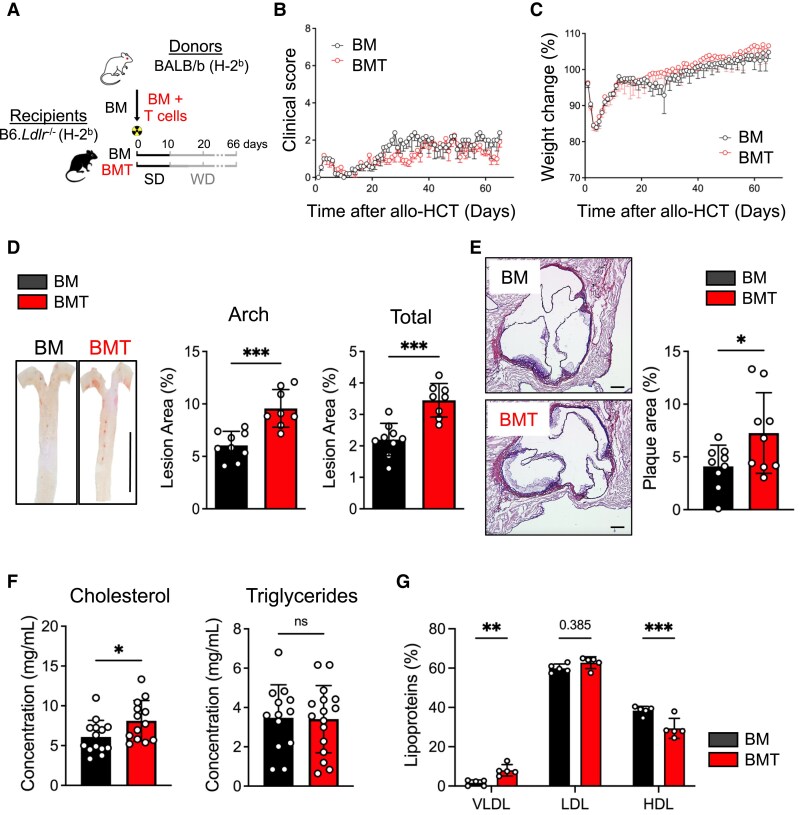
Subclinical acute GvHD aggravates atherosclerosis. (*A*) Experimental design of the miHAg-mismatched allo-HCT-induced atherosclerosis model. After myeloablative irradiation (9 Gy), miHAg-mismatched allogeneic 8–12-week-old male B6.*Ldlr*^−/−^ (H-2b) recipients were transplanted either with 5 × 10^6^ BM cells only (BM group, *n* = 15) or 5 × 10^6^ BM cells and 5 × 10^6^ enriched T cells (BMT group, *n* = 16) from 8- to 12-week-old BALB/b (H-2b) donors (BALB/b → B6.*Ldlr*^−/−^). Starting on Day 10 after HCT, B6.*Ldlr*^−/−^ recipient mice were fed with WD for 8 weeks. (*B*) Clinical GvHD score and (*C*) body weight change calculated as the percentage of the weight at the beginning of the experiment. (*D*) Quantification of Oil-Red-O-stained aortas. Enface images of the aorta, scale bar 1 cm. (*E*) Representative images and quantification of plaque area in Aldehyde-Fuchsin-stained aortic root sections; scale bar, 100 μm. (*F*) Quantification of cholesterol and triglycerides in serum. (*G*) Quantification of VLDL, LDL, and HDL in serum. Combined data from two independent experiments are shown. Statistical significance was determined by unpaired non-parametric Mann–Whitney test. Data are presented as a mean ± std dev. *P < 0.05, **P < 0.01, ***P < 0.001. ns, non-significant.

To address whether GvHD and atherosclerosis are interconnected, we established a major histocompatibility complex-matched, miHAg-mismatched allo-HCT mouse model. GvHD can occur when the host and donor are incompatible, particularly when residual T cells are present in the BM graft or when donor T cells expand following successful BM engraftment. In mice, BM is enriched in regulatory T cells (Tregs),^36^ necessitating the co-transfer of splenic T cells from mismatched donors along with BM to induce GvHD. To model this, we transplanted mice of the C57BL/6 (B6) background with BM, or BM and CD3^+^ T cells (BMT) from (BALB/b) mice (BALB/b (H-2^b^) → B6 (H-2^b^)). In our study, B6 recipients were additionally deficient in *Ldlr*^−/−^, and in combination with a WD, therefore more susceptible to atherosclerosis. Starting at 10 days after allo-HCT with BALB/b BM or BMT, we fed B6.*Ldlr*^−/−^ recipients with a standard diet (SD) in a control experiment or a WD and monitored them over 8 weeks. B6.*Ldlr*^−/−^ recipients transplanted with BM or BMT and fed a SD did not show alterations in their survival or clinical signs of GvHD (see Supplementary material online, *Figure S1A–C*). Mice receiving the WD developed mild clinical GvHD scores around Day 20 after allo-HCT (*Figure 1A* and *B*) without changes in survival and relative body weight (see Supplementary material online, *Figure S1A* and *Figure 1C*) when comparing BM- or BMT-transplanted groups. Next, we examined atherosclerotic lesion formation at two typical predilection sites in the aorta and aortic root. As expected, only marginal atherosclerotic lesions formed in SD-fed BM and BMT-transplanted mice in the aorta and the aortic root (see Supplementary material online, *Figure S1D* and *E*). In contrast, male and female mice fed with WD developed early atherosclerotic lesions in the aortic arch and total aorta, as well as in the aortic root, which were increased in BMT-transplanted mice compared with mice transplanted with BM only (*Figure 1D* and *E* and Supplementary material online, *Figure S2*). In contrast, the histopathological scoring of GvHD target organs, liver, colon, small intestine, and skin showed only mild tissue injury and no differences between BM- and BMT-transplanted mice (see Supplementary material online, *Figure S3*). Notably, the BMT group exhibited significantly elevated serum cholesterol levels compared with BM only recipients, while triglyceride levels remained unchanged (*Figure 1F* and Supplementary material online, *Figure S2*). To further assess lipid distribution, we analysed cholesterol levels within very low density lipoprotein (VLDL), LDL, and high density lipoprotein (HDL) in serum. Whereas LDL-cholesterol levels were similar, significantly elevated VLDL cholesterol levels and decreased HDL cholesterol levels were noted in BMT recipients (*Figure 1G*), indicating an overload of ApoB-protein-containing lipoproteins in BMT mice. These data demonstrate aggravated atherosclerosis and increased cholesterol levels in mice transplanted with BMT compared with BM only, even in the absence of overt clinical GvHD or disease activity in acute GvHD target organs. This suggests that subclinical GvHD acts as a WD-related and organ-specific disease trigger in both male and female mice.

To determine whether syngeneic T-cell responses could induce similar pathology, we performed syngeneic-HCT (syn-HCT) by transplanting B6.*Ldlr*^−/−^ mice with BM, or BM and CD3^+^ T cells (BMT) from B6 mice. Starting at 10 days after syn-HCT, we fed recipients with a WD and monitored them over 8 weeks, mirroring our allo-HCT approach (see Supplementary material online, *Figure S4A* and *B*). Unlike in the allo-HCT model, syngeneic BM and BMT recipients exhibited no differences in atherosclerotic lesion formation within the aortic arch, total aorta, or aortic root (see Supplementary material online, *Figure S4C* and *D*). Additionally, cholesterol levels remained comparable between these groups (see Supplementary material online, *Figure S4E*). Plaques in these experiments were larger than those observed in allo-HCT-transplanted mice. This may be attributed to the BALB/b background of transplanted cells in the allogenic model that—congenic with the BALB/c strain—typically produces smaller atherosclerotic lesions compared with B6 mice.^37,38^ These results demonstrate that aggravated atherosclerotic lesion formation and increased serum cholesterol levels were specifically driven by allogeneic T cells in our model.

### 3.2 Subclinical GvHD locally affects aortic T cells in atherosclerosis

**Figure 2:**
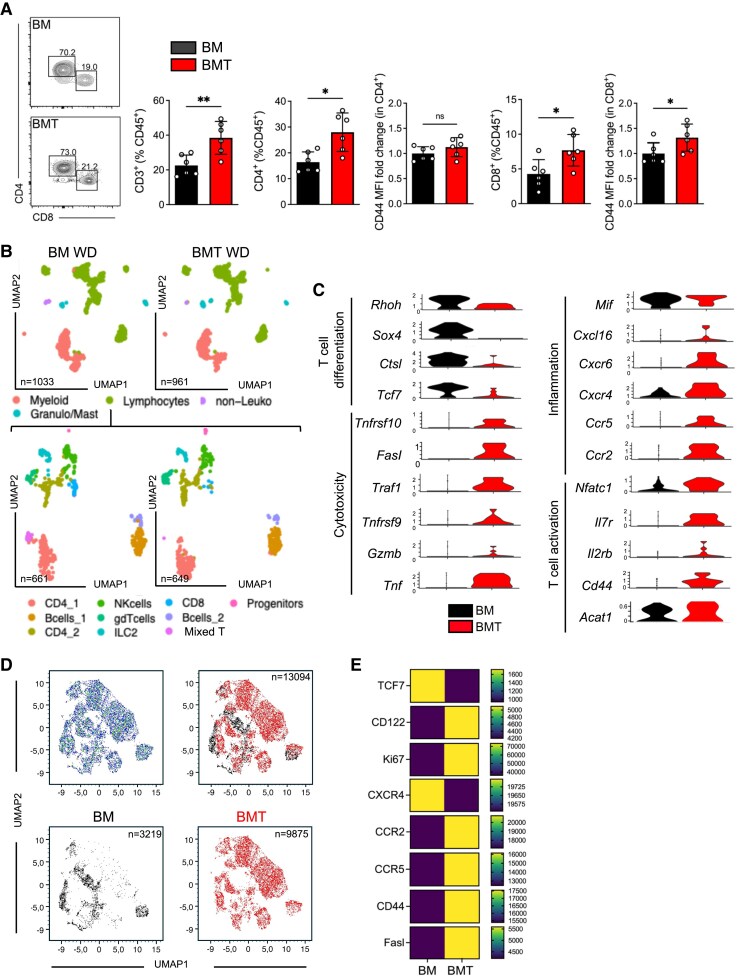
GvHD increases CD8^+^ T-cell percentage and activation in aorta after 8 weeks WD feeding. miHAg-mismatched allogeneic male B6.*Ldlr*^−/−^ (H-2b) recipients were transplanted either with 5 × 10^6^ BM cells only (BM group) or 5 × 10^6^ BM cells and 5 × 10^6^ enriched T cells (BMT group) from BALB/b (H-2b) donors (BALB/b → B6.*Ldlr*^−/−^) and fed a WD starting 10 days after HCT for 8 weeks. (*A*) Gating of CD4^+^ and CD8^+^ T cells in the aorta of in BMT compared with BM group (pre-gated on singlet/viables/CD3ε^+^). Percentage of total CD3^+^ T cells, CD4^+^ T cells, and CD8^+^ T cells relative to total leucocytes in the aorta in BMT compared with BM group. Mean fluorescence intensity (MFI) of CD44 expression on CD4^+^ and CD8^+^ T cells presented as fold change. (*B*) Uniform Manifold Approximation and Projection (UMAP) of aligned gene expression data in aortic single cells of BM (left) and BMT (right) group demonstrating total cells (up) and re-clustered lymphocytes (bottom). (*C*) Expression of genes in CD8^+^ T cells indicative for T-cell activation, inflammation, cytotoxicity and T cells differentiation comparing BM and BMT group. (*D*) UMAP generated by FlowJo using UMAP package by protein expression pattern (Ki67, CD127, Fasl, CXCR4, CCR2, FoxP3, CD44, CCR5, TNFα, Granzyme B, TCF7, CD122) in aortic single cells of BM and BMT groups, and concatenated BM and BMT. (*E*) Expression of CD122, Ki67, CXCR4, CCR2, and CCR5 (MFI) in concatenated CD8^+^ T cells from BM and BMT groups. Data are presented as mean ± std dev. Statistical significance was determined by unpaired non-parametric Mann–Whitney test. *P < 0.05, **P < 0.01. ns, non-significant; Granulo/Mast, Granulocytes/Mast cells; non-Leuco, non-Leucocytes; NK cells, Natural killer cells.

To gain further insight into the cellular plaque composition and phenotype, atherosclerotic lesions were analysed by immunohistochemistry. We could not detect changes in the relative content of plaque Mac2^+^ macrophages and αSMA^+^ vascular smooth muscle cells by immunostaining or in collagen content and necrotic core size in the aortic root (see Supplementary material online, *Figure S5*). However, the frequencies of CD3^+^ T cells, comprising both CD4^+^ and CD8^+^ T cells, were elevated in the aorta of mice transplanted with BMT compared with BM-recipients (*Figure 2A*). Furthermore, aortic CD8^+^ T cells but not CD4^+^ T cells displayed an increased expression of CD44 in BMT recipients (*Figure 2A*), indicative of an effector phenotype. To further characterize the phenotype of lesioned immune cells, we performed scRNA-seq analyses of isolated total CD45^+^ cells from the aorta of B6.*Ldlr*^−/−^ mice transplanted with BM or BMT and fed a WD for 8 weeks. Unsupervised clustering identified distinct clusters of myeloid cells (*CD68*, *Trem2*, *Ccr2*, and *Nlrp3*) granulocytes/mast cells (*S100a8*, *Il1rl1*), lymphocytes (*Cd3e*, *Cd19*, *Ccr7*, and *Cd27*), and few contaminating non-leucocytes (*Figure 2B* and Supplementary material online, *Figure S6A*). We selected lymphocytes for re-clustering and could discriminate different subclusters of CD4^+^ and CD8^+^ T cells, B cells, ɣδT cells, ILC2s, non-leucocytes (NKs) cells, and a mixed T-cell cluster containing both CD4^+^ and CD8^+^ T cells (*Figure 2B* and Supplementary material online, *Figure S6B* and *S7A*). Based on our observation of an expanded population of CD8^+^ T cells in mice transplanted with BMT, we analysed the gene ontology of this cell subset, even with limited cell numbers in this cluster. CD8^+^ T cells from mice receiving BMT were enriched for biological processes, including cellular response to cytokine stimulus, whereas CD8^+^ T cells from BM-transplanted mice were enriched with putative functions of e.g. cell differentiation (see Supplementary material online, *Figure S6C* and *D*). In line with flow cytometric analyses, CD8^+^ T cells from BMT-transplanted mice showed significantly increased expression levels of cell activation markers, e.g. *Nfatc1, Il7r*, *Il2rb*, and *Cd44* (*Figure 2C*). Moreover, CD8^+^ T cells from BMT upregulated genes associated with cytotoxic T-cell differentiation (such as *Tnfrsf9* and *Traf1*) and cytotoxic function (including *Gzmb*, which encodes granzyme B, *Fasl*, *Tnfsf10*) as well as the inflammatory cytokine *Tnf* and several chemokine receptors (*Ccr2*, *Ccr5*, *Cxcr4*, and *Cxcr6)* (*Figure 2C*). In contrast, CD8^+^ T cells from BM-recipients expressed higher levels of differentiation-related genes (such as *Tcf7*, *Sox4*). To further validate these findings at the protein level, we performed spectral flow cytometry on CD8^+^ T cells isolated from the aortas of *Ldlr*^−/−^ mice that had received either BM or BMT and were fed a WD for 8 weeks. Analysis of 13 094 concatenated CD8^+^ T cells (3219 cells from BM and 9874 cells from BMT groups) revealed a marked increase in the expression of granzyme B, tumour necrosis factor alpha (TNFα), FasL, CD44, and other effector markers in the BMT group compared with the BM group (*Figure 2D* and *E* and Supplementary material online, *Figure S7B* and *C*). These results confirm that CD8^+^ T cells in the BMT group showed a higher activation and cytotoxicity state.

Next, we evaluated systemic immune responses. White blood counts (WBC), spleen weight, or mesenteric and peripheral LN cell counts did not differ between groups (see Supplementary material online, *Figure S8A–C*). Similarly, total monocyte levels and counts of Ly6C^high^ and Ly6C^low^ monocytes as precursors of plaque macrophages remained unaltered in blood and spleen between groups (see Supplementary material online, *Figure S8*). Furthermore, both groups presented mostly similar total B and T cells in blood, spleen, and LNs, similar frequencies of CD4^+^ and CD8^+^ T cells among CD3^+^ T cells, and similar differentiation into naïve, effector, and central memory T cells (see Supplementary material online, *Figure S9*), as well as similar frequency of Foxp3^+^ CD25^+^ CD4^+^ regulatory T cells (Tregs, data not shown) except for lower Treg frequencies in mesenteric and peripheral LNs (see Supplementary material online, *Figure S9D–G*).

Dysfunctional vascular endothelial cells play a crucial role in atherosclerosis by LDL accumulation and inflammation—all of which promote plaque formation. Additionally, the vascular endothelium can function as non-haematopoietic antigen-presenting cells (APCs) by activating allogeneic CD8^+^ T cells through CD80-dependent co-stimulation,^39^ even in the absence of conventional haematopoietic APCs.^40^ In our study, we investigated the impact of myeloablative irradiation on the vascular endothelium, focusing on the expression of major histocompatibility complex class I (MHCI) and integrin subunits that regulate immune responses and tissue infiltration. Under steady-state conditions, the vascular endothelium exhibited high levels of MHCI, which were modestly down-regulated following myeloablative irradiation. Conversely, irradiation induced a time-dependent upregulation of alpha integrins CD49b (integrin α2), CD49d (integrin α4), and CD49e (integrin α5), suggesting an enhanced capacity for immune cell recruitment (see Supplementary material online, *Figure S10*).

These data demonstrate that increased lesion formation in mice transplanted with BMT correlated with an increased infiltration of activated CD8^+^ T cells locally in the aorta, accompanied by an expansion of conventional T cells and a decrease in CD4^+^ Tregs in peripheral and mesenteric LNs.

### 3.3 Subclinical GvHD-atherosclerosis is not associated with liver injury, but an upregulation of pathways to lower cholesterol

**Figure 3:**
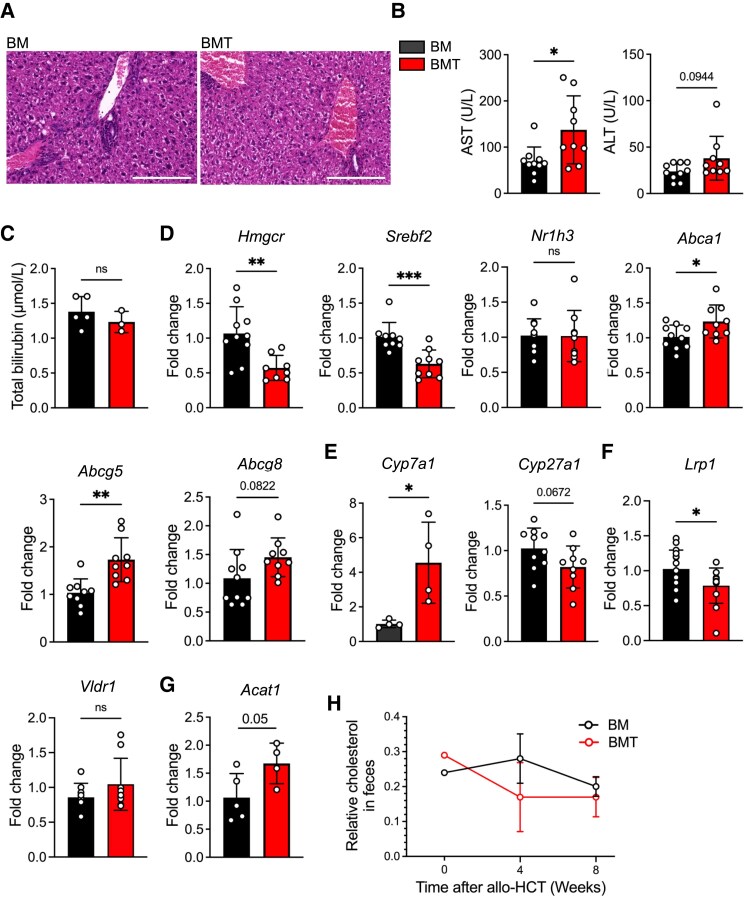
Changes in the liver during subclinical GvHD. miHAg-mismatched allogeneic male B6.*Ldlr*^−/−^ (H-2b) recipients were transplanted either with 5 × 10^6^ BM cells only (BM group) or 5 × 10^6^ BM cells and 5 × 10^6^ enriched T cells (BMT group) from BALB/b (H-2b) donors (BALB/b → B6.*Ldlr*^−/−^) and fed a WD starting 10 days after HCT for 8 weeks. (*A*) HE-stained liver sections; scale bar, 200 µm. (*B*) AST and ALT levels in serum. (*C*) Total bilirubin level in serum. (*D*–*G*) Relative mRNA expression of indicated genes in the liver. (*H*) Cholesterol levels in faeces. Data are presented as a mean ± std dev. Statistical significance was determined by unpaired non-parametric Mann–Whitney test. *P < 0.05. ns, non-significant.

As the liver plays a critical role in controlling cholesterol metabolism and is a primary target organ for GvHD, we examined the phenotype of the liver after allo-HCT. Histopathological scoring of the liver revealed mild regional signs of regeneration, stellate cell activation, and lobular and portal inflammation but no differences between BM- and BMT-transplanted mice (*Figure 3A* and Supplementary material online, *Figure S3*). Conversely, serum levels of the liver enzyme aspartate transaminase (AST) were significantly elevated, and alanine transaminase (ALT) showed a mild increase, indicating liver damage (*Figure 3B*); total bilirubin was unaltered between groups (*Figure 3C*). These changes in liver enzymes were not observed after syn-HCT in BM and BMT groups (see Supplementary material online, *Figure S4F*).

As BMT-transplanted mice showed increased serum cholesterol levels, we further evaluated the gene expression of genes regulating cholesterol production and disposal. The expression of *Hmgcr* encoding 3-hydroxy-3-methylglutaryl–coenzyme A (HMG-CoA) reductase, the rate-limiting gene-regulating cholesterol synthesis, and the transcription factor *Srebf2* controlling cholesterol synthesis by regulating sterol-regulated genes was decreased in BMT vs. BM-transplanted mice. The expression of the liver X receptor (LXR, encoded by *Nr1h3*) was unaltered. However, examining cellular transporters removing cholesterol from the cells, we found a significant increase in *Abca1* and *Abcg5,* and a trend towards an increase in *Abcg8* mRNA expression in BMT-transplanted mice (*Figure 3D*). Moreover, the expressions of hydroxylases, which are important for the synthesis of bile acids from cholesterol via the classical (*Cyp7a1*) but not the alternative pathway (*Cyp27a1*), were increased in BMT- vs. BM-recipients (*Figure 3E*). Taken together, these data indicate mild liver damage and activation of compensatory pathways to lower cholesterol levels in BMT-transplanted mice.

ApoB lipoproteins can be taken up by Kupffer cells (KCs) in the liver, which then secrete factors that modulate atherosclerosis.^41^ However, none of the KC target genes identified in response to ApoB dyslipidaemia^41^ (*C6*, *Folr2*, *Grn*, *Il18bp*, *Sdc3*, *Ifrd1*, *Nop58*, and *Socs2*) were significantly altered between groups, and *Themis2* was even down-regulated in BMT-transplanted mice (see Supplementary material online, *Figure S11A*). Yet, an increase in the inflammatory cytokines *Il6* and *Ccl2* but not *Tnf* and *Il1b* was noted in BMT recipients (see Supplementary material online, *Figure S11B*).

Therefore, we next examined the expression of other lipoprotein receptors involved in clearing chylomicron remnants, which carry primarily cholesterol and cholesterol esters, and found significantly reduced expression of LDL receptor-related protein 1 (*Lrp1*). In contrast, the expression of the VLDL receptor (*Vldr1*) remained unchanged (*Figure 3F*). *Acat1*, which encodes acetyl-CoA acetyltransferase 1 and is involved in the production of lipoproteins like LDL and VLDL, showed a trend towards upregulation in the liver (*Figure 3G*). In contrast, expression of hepatic lipase activity (*Lipc*), which hydrolyses triglycerides and thereby converts intermediate-density lipoprotein (IDL) to LDL, of lipoprotein lipase (*Lpl*), hydrolyzing triglycerides in chylomicrons, as well as endothelial lipase G (*Lipg*), involved in HDL catabolism, were unaltered (see Supplementary material online, *Figure S11C*). This suggests an increase in hepatic lipoprotein formation and VLDL secretion alongside a reduced uptake of lipoproteins.

Interestingly, cholesterol levels in faeces were comparable between BMT- vs. BM-recipients (*Figure 3H*), suggesting no significant differences in cholesterol loss or reabsorption through the intestine. In summary, the combination of reduced *Lrp1* expression and the trend towards elevated *Acat1* expression in the livers of BMT-transplanted mice likely contributed to the overall increase in serum cholesterol levels.

### 3.4 Depleting CD8^+^ T cells ameliorated allo-HCT-induced atherosclerotic plaque formation

**Figure 4:**
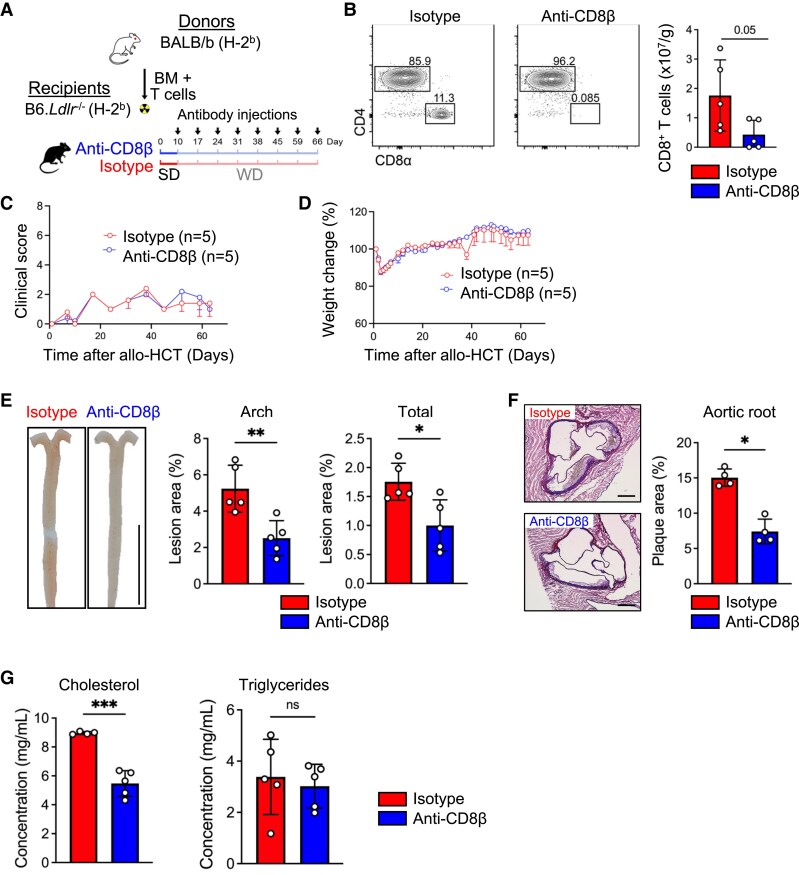
CD8^+^ T-cell depletion prevents plaque formation in aortic root and aorta in the subclinical GvHD model. (*A*) Experimental design. After myeloablative irradiation (9 Gy), miHAg-mismatched allogeneic 8–12 weeks old male B6.*Ldlr*^−/−^ (H-2b) recipients were transplanted either with 5 × 10^6^ BM cells only (BM group, *n* = 15) or 5 × 10^6^ BM cells and 5 × 10^6^ enriched T cells (BMT group, *n* = 16) from 8 to –12 weeks old BALB/b (H-2b) donors (BALB/b → B6.*Ldlr^−/−^*). Starting on Day 10 after HCT, B6.*Ldlr*^−/−^ recipient mice were fed with WD for 8 weeks, and treated with anti-CD8β or isotype control antibodies (i.p.) once per week (*n* = 5). (*B*) Representative flow cytometry dot plots and quantification of CD8^+^ T cells in spleen. (*C*) Clinical GvHD score. (*D*) Relative body weight change. (*E*) Quantification of Oil-Red-O-stained aortas. Enface images of the aorta; scale bar, 1 cm. (*F*) Representative images and quantification of plaque area in Aldehyde-Fuchsin-stained aortic root sections; scale bar, 100 μm. (*G*) Quantification of cholesterol and triglycerides levels in serum. Data are presented as a mean ± std dev. Statistical significance was determined with unpaired non-parametric Mann–Whitney test, *P < 0.05, **P < 0.01, ***P < 0.001.

In patients receiving donor lymphocyte infusion (DLI), CD8^+^ T-cell depletion decreases the incidence of GvHD.^9–11^ In atherosclerosis, we and others have previously demonstrated a pro-atherosclerotic role of CD8^+^ T cells in early lesion formation in *Ldlr*^−/−^ and apolipoprotein E-deficient mice.^21,22^ Given that the enhanced atherosclerotic lesion formation in BMT-transplanted mice was associated with increased proportions of activated allogeneic CD8^+^ T cells displaying heightened cytotoxicity in the aorta, we assessed the role of CD8^+^ T cells in atherosclerotic lesion formation in our model. To this end, B6.*Ldlr*^−/−^ mice transplanted with BALB/b BMT were treated with anti-CD8β monoclonal antibody or an isotype-matched control antibody over 8 weeks, starting with the WD at Day 10 after transplantation (*Figure 4A*). When we euthanized BMT recipients after 8 weeks, we confirmed decreased numbers of CD8^+^ T cells in the spleen in anti-CD8β compared with isotype antibody-treated mice (*Figure 4B*). Survival, GvHD clinical score, and relative body weight did not differ between anti-CD8β compared with isotype antibody-treated BMT-transplanted mice (*Figure 4C* and *D* and Supplementary material online, *Figure S12A*), similar to prior experiments. Nonetheless, the decrease in CD8^+^ T cells led to a reduction in atherosclerotic lesion formation in the aortic arch, total aorta, and in the aortic root (*Figure 4E* and *F*). The plaque phenotype did not show differences in relative macrophage or collagen content, but a trend towards increased smooth muscle cells (SMC) content and smaller necrotic cores in mice treated with anti-CD8β antibody (see Supplementary material online, *Figure S12B–E*), suggestive of a less advanced and more stable plaque phenotype, as also reflected by a decrease in the plaque vulnerability index (see Supplementary material online, *Figure S12F*). Of note, CD8^+^ T-cell depletion in B6.*Ldlr*^−/−^ mice transplanted with BALB/b BM only reduced atherosclerotic lesion formation in the aorta but not the aortic arch and aortic root (see Supplementary material online, *Figure S13*), suggesting that CD8^+^ T cells in part or site-specifically promote atherosclerotic lesion formation via allogeneic CD8^+^ T-cell responses but also CD8^+^ T effector functions previously observed in early atherosclerosis.^21,22,42^ In contrast, cholesterol levels were decreased in BMT, but not BM-transplanted mice treated with anti-CD8β compared with control antibody without alterations in the levels of triglycerides (*Figure 4G* and Supplementary material online, *Figure S13*), demonstrating that allogeneic CD8^+^ T-cell responses contribute to increased cholesterol levels in mice transplanted with BMT.

## 4. Discussion

In this study, we devised a novel mouse model to investigate a potential interconnection between allo-HCT and atherosclerosis. We discovered that the allogeneic miHAg-mismatched transplantation of BALB/b donor BMT into atherosclerosis-prone C57BL/6 *Ldlr*^−/−^ mice, even without causing overt clinical signs of acute GvHD, accelerated atherosclerotic lesion formation and increased serum cholesterol levels compared with recipient C57BL/6 *Ldlr*^−/−^ mice reconstituted with BALB/b donor BM only. This phenotype was not observed upon syn-HCT. Our experiments using antibody-mediated depletion of CD8^+^ T cells furthermore demonstrated that this phenotype was at least in part dependent on donor CD8^+^ T cells. These data provide evidence that even in subclinical GvHD, allo-reactive donor CD8^+^ T cells aggravate hypercholesterolaemia and atherosclerosis.

Most mouse strains are relatively resistant to the development of atherosclerosis. Under conditions of hypercholesterolaemia, however, e.g. induced by WD feeding of *Ldlr*^−/−^ mice (Bl6.*Ldlr*^−/−^), which cannot efficiently clear non-HDL lipoproteins from the blood, these mice develop atherosclerotic lesions at typical predilection sites in the aortic root and aorta.^43^ Here, we transplanted B6.*Ldlr*^−/−^ mice with BALB/b donor BM with/without T cells and started WD feeding at 10 days after the transplantation, when the irradiation-induced damage of the intestinal barrier has subsided.^27^ C57BL/6 mice are more susceptible to the development of atherosclerosis than the BALB/c strain.^38^ BALB/b mice are congenic with the BALB/c strain.^37^  *Ldlr*^−/−^ mice fed a SD after BM/BMT transplantation did not develop noticeable atherosclerotic lesions, as expected.^43^ WD-fed *Ldlr*^−/−^ mice transplanted with allogeneic BMT developed larger atherosclerotic lesions in the aortic root and aorta compared with the lesions in mice transferred with BM. Vascular lesions formed even in the absence of strong clinical symptoms of GvHD or major histopathological changes in GvHD target organs skin, liver, small intestine, and colon, suggesting that vascular changes may represent a WD-induced, subclinical form of GvHD in the aorta, driven by allogeneic CD8^+^ T-cell responses.

A striking finding was the increase in serum cholesterol and VLDL levels in BMT compared with BM-transplanted mice, and a reduction in serum cholesterol levels in BMT-transplanted mice depleted of CD8^+^ T cells, indicating that subclinical GvHD in our model was associated with aggravated hypercholesterolaemia, which was at least partly dependent on CD8^+^ T cells. Cholesterol homeostasis is a tightly regulated process, with serum LDL-cholesterol being regulated by reciprocal crosstalk between intestinal absorption of dietary and biliary cholesterol, as well as hepatic biosynthesis of cholesterol and clearance.^44^ Both the intestine and liver are key GvHD target organs.^26^ Although histological analyses did not reveal major pathological changes or differences between BM- and BMT-transplanted mice, there was a slight tendency towards increased levels of transaminases in BMT-transplanted mice, suggesting mild liver damage. However, the expression of genes involved in cholesterol homeostasis indicated the induction of a counter-regulatory mechanism to lower cholesterol levels: HMG-CoA expression (encoded by *Hmgcr*), the rate-controlling enzyme in the cholesterol synthesis pathway, and SREBP2 (sterol regulatory element-binding protein, encoded by *Srebf2*), involved in cholesterol synthesis, were decreased in BMT compared with BM-transplanted mice. In addition, cytochrome P450 7a1 (*Cyp7a1*), involved in controlling cholesterol conversion into bile acids, and ABCG5, which together with ABCG8 is required for the excretion of hepatic cholesterol into bile, were increased. Hyperlipidaemia is frequently observed in patients with chronic cholestatic liver disease, and in acute GvHD, cholestasis is caused by the damage of the small bile ducts and often associated with hyperbilirubinaemia.^26^ We did not observe changes in serum bilirubin levels, which, together with the absence of damage in the histopathological evaluation of the liver, suggest alternative mechanisms underlying the increase in serum cholesterol levels or an early disease stage not yet detectable by histology. However, reversal of cholesterol levels in BMT-transplanted mice depleted of CD8^+^ T cells strongly suggest CD8^+^ T-cell-dependent effects.

Our study demonstrates that BMT transplantation significantly disrupts lipoprotein metabolism in the context of metabolically challenged mice and hepatic cholesterol regulation. In addition to the expected increase in LDL due to LDLR deficiency, the BMT group displayed markedly elevated VLDL and reduced HDL levels, suggesting an overload of ApoB-containing lipoproteins in circulation. Recent studies have suggested that excessive ApoB lipoproteins can be taken up by KCs in the liver,^41^ triggering the secretion of factors that modulate atherosclerosis. Consistent with these findings, our data indicate that the liver adapts to increased intracellular cholesterol levels by downregulating intrahepatic enzymes involved in cholesterol biosynthesis, such as *Srebf2* and *Hmgcr*, and upregulating those that reduce intracellular cholesterol, including *Cyp7a1*, *Abca1*, and *Abcg5*. Notably, the expression of known KC target genes^41^ did not differ significantly between BM and BMT groups, suggesting that additional mechanisms may be involved. Further analysis revealed a notable reduction in *Lrp1* and increased *Acat1* expression in the livers of BMT-transplanted mice. A decreased LRP1 expression likely impairs hepatic clearance of lipoproteins, in particular in the absence of LDLR, while the elevated *Acat1* expression may enhance intracellular cholesterol esterification and VLDL secretion. We observed no major differences in faecal cholesterol excretion between groups. Collectively, these alterations likely contributed to the increased serum cholesterol levels observed in the BMT group. Future studies should investigate the role of T cells in modulating hepatic cholesterol metabolism, particularly their effects on lipoprotein receptor expression. Regardless of the mechanistic underpinnings, elevated cholesterol levels remain critical drivers of atherosclerotic lesion formation, which likely contributed to the enhanced lesion development seen in BMT-transplanted mice in our study. Given the absence of LDLR in *Ldlr*^−/−^ mice, and therefore their inability to upregulate LDLR in response to low hepatic cholesterol levels or inflammation, further studies are necessary to investigate the development of atherosclerosis and cholesterolaemia in patients after allo-HCT and in mice with different genetic backgrounds.

Allogeneic T cells that expand and attack recipient cells are the primary drivers of GvHD, whereas Tregs mitigate disease severity.^33,45–50^ Our analysis of immune cells in various organs revealed a reduction Tregs in mesenteric and inguinal LNs. Tregs numbers in the aorta are low,^51^ and we did not detect any changes in their frequencies in the aorta by flow cytometry. A reduction of atheroprotective Tregs^52^ systemically, however, may have contributed to an increased atherosclerotic lesion formation in our study. We, in addition, noted an increased proportion of activated CD8^+^ T cells in the atherosclerotic aorta and an expansion of T cells in LNs. Although there were no changes in the number of T cells in blood or alterations in the proportion of T cells in spleen, this suggests T-cell activation in LNs and the specific accumulation of activated CD8^+^ T cells in the aorta. scRNA-seq revealed striking differences in gene expression related to T-cell activation and cytotoxic effector functions in CD8^+^ T cells from BMT vs. BM-transplanted mice. For instance, genes associated with the induction of apoptosis of target cells through the granule secretory pathway involving granzyme B and the TNF family member FasL were enriched in CD8^+^ T cells from BMT-transplanted mice. These mediators drive cytotoxicity of CD8^+^ T cells towards different plaque cell types^22,53^ and promote necrotic core formation and plaque vulnerability in atherosclerosis.^21,22^ Furthermore, we detected an increased expression of *Nfatc1*, which controls the cytotoxicity of CD8^+^ T cells^54^ and promotes GvHD by amplifying the proliferation, target tissue homing, and effector functions of allogeneic donor T cells.^49^

Notably, our findings align with the notion that CD8^+^ T cells are particularly sensitive to cholesterol levels. Prior studies have shown that cholesterol can amplify CD8^+^ T-cell responses,^55,56^ promoting their proliferation and the production of cytotoxic molecules and pro-inflammatory cytokines—a phenomenon we observed in the BMT group. Moreover, these aortic CD8^+^ T cells were not only expanded but also displayed a heightened inflammatory and cytotoxic phenotype compared with those from BM-transplanted mice, potentially contributing to enhanced atherosclerotic lesion formation in our GvHD-atherosclerosis model. Importantly, our data also suggest that allogeneic CD8^+^ T cells drive disease in the liver as a GvHD target organ, thereby raising cholesterol levels. This is supported by our finding that depletion of CD8^+^ T cells using an anti-CD8β antibody significantly ameliorated the BMT-induced increase in cholesterol levels but did not affect cholesterol levels in mice transplanted with BM only. Both pathomechanisms likely contribute to the augmented atherosclerotic lesion formation in hypercholesterolaemic mice with chronic GvHD. While an increased incidence of cardiovascular disease after allo-HCT constitutes a major clinical problem requiring alertness and preventive care,^57^ it is still controversial whether GvHD is the underlying primary driver. This study supports the notion that allo-HCT contributes to the increase in atherosclerotic lesion burden, while also uncovering CD8^+^ T-cell effects independent of BMT transplantation, a phenomenon similarly observed in non-transplanted *Ldlr*^−/−^ mice during early atherosclerosis.^21,22^  ^,42^

Prior studies showed that tissue damage is a prerequisite for the accumulation of donor CD8^+^ T cells in GvHD, and that CD8^+^ T-cell-dependent GvHD requires direct interactions between donor T cells and target cells.^58^ It is conceivable that an enhanced lipid deposition instigated the early recruitment of immune cells to the vessel wall to promote early lesion formation and local tissue damage and that effector functions of donor CD8^+^ T cells towards antigen-mismatched recipient cells of the artery wall and also atherosclerosis-relevant antigens^59^ then colluded locally to amplify inflammation and atherosclerotic lesion growth specifically in the arterial vessel wall already affected by atherosclerosis.

By establishing a novel mouse model, we uncovered that allo-HCT and minor mismatch transplantation of atherosclerosis-prone Bl6.*Ldlr*^−/−^ mice with BALB/b donor BMT accelerated atherosclerotic lesion formation and increased serum cholesterol levels. Furthermore, we could identify a critical role of CD8^+^ T cells herein and the reduction of the allo-HCT-induced increase in atherosclerotic lesion formation and cholesterol levels in mice depleted of CD8^+^ T cells. These data may provide a novel mechanistic underpinning for the clinically observed increased cardiovascular disease in long-term allo-HCT survivors,^1,2,5,6^ and present an opportunity to utilize this mouse model for further research on the signalling pathways involved in GvHD-induced atherosclerosis, potentially leading to the identification of innovative therapeutic approaches.

## Supplementary Material

cvaf229_Supplementary_Data

## Data Availability

Single-cell RNA sequencing data have been uploaded to the Gene Expression Omnibus database under accession ID GSE291588. Other raw data underlying this article are available upon request.
