# Supplementary material for: Allogeneic haematopoietic cell transplantation promotes atherosclerosis in mice via CD8+ T cells
Source: Cardiovasc Res. 2025 Nov 18;121(17):2668–78. doi: 10.1093/cvr/cvaf229 (PMC12754844; doi:10.1093/cvr/cvaf229)
Supplement: cvaf229_Supplementary_Data [file cvaf229_supplementary_data.pdf]

SUPPLEMENTAL DATA

Allogeneic hematopoietic cell transplantation initiates atherosclerosis in mice via CD8$^+$ T cells

Ivana Jorgacevic, Haroon Shaikh, Hla Ali, Maja Bundalo, Sarah Schäfer, Michael A.G. Kern, Maike Büttner-Herold, Simone Reu-Hofer, Clément Cochain, Hendrik Bartolomaeus, Antoine-Emmanuel Saliba, Melanie Rösch, Giuseppe Rizzo, Estibaliz Arellano Viera, Juan Gamboa Vargas, Friederike Berberich-Siebelt, Wolfgang Herr, Louis Boon, Andreas Rosenwald, Elke Butt, Heike M. Hermanns, Andreas Beilhack, Alma Zernecke

Detailed Materials and Methods

Mice and allo-HCT

Experiments were performed with 8 to 12-week-old male or female mice in a sex-matched miHAg mismatch BALB/b (H-2Kb) → C57BL/6.*Ldlr*$^{-/-}$ (H-2Kb) model. C57BL/6.*Ldlr*$^{-/-}$ (B6.*Ldlr*$^{-/-}$) and BALB/b mice were purchased from Charles River Laboratory (Sulzfeld, Germany) or bred locally at the animal facility at the Centre of Experimental Molecular Medicine (ZEMM), University Hospital Würzburg, Germany. For allo-HCT, B6.*Ldlr*$^{-/-}$ recipients were myeloablatively irradiated with 9 Gy and intravenously injected with 5x10^6 BALB/b donor bone marrow (BM) with/without 5x10^6 WT splenic T cells. T cells were enriched by negative selection from the spleen of 8- to 12-week-old donor BALB/b mice by using Dynabeads™ Untouched™ Mouse T Cell Kit (Thermo Fisher Scientific) according to the manufacturer's protocol. T cell purity reached 85–95%, as assessed by flow cytometry.

To induce atherosclerosis, recipient mice were placed on a WD (21% fat, 0,15% cholesterol, altromin) starting 10 days after the allo-HCT. After 8 weeks of WD, animals were euthanized by an overdose of isoflurane anesthesia (5% concentration) with subsequent cervical dislocation, exsanguination and organ extraction. A group of mice received once weekly intraperitoneal injections of 250 µg rat anti-mouse–CD8β IgG2b antibody (Ab) (clone YTS156.7.7, referred as anti-CD8β) or isotype-matched irrelevant rat anti-Phyt1 (clone AFRC-MAC51, referred to as control), starting on day 10 post allo-HCT. All animal studies conformed to the Directive 2010/63/EU of the European Parliament and had been approved by the appropriate local authorities (Regierung von Unterfranken, Würzburg, Germany).

GvHD scoring

Mice were scored daily for the first seven days after allo-HCT and then three times per week unless a score threshold was met, which triggered more frequent assessments. The score evaluated four key areas: body weight (body weight loss thresholds ranged from >10% to >25%), fur condition (ranging from slight roughness to widespread shagginess), posture (from mild kyphosis to severe mobility restriction), and activity (from reduced movement to complete immobility). Points were assigned based on severity, with cumulative scores dictating interventions. Mild stress required increased monitoring, moderate stress supportive measures such as softened food and more frequent checks, while severe stress mandated immediate euthanasia. While transient weight loss is expected early post-allo-HCT, GvHD

symptoms typically emerge by the third week onwards, including fur changes, kyphosis, and activity reduction.

Atherosclerotic lesion quantification and immunohistochemistry
Arteries were flushed with PBS and perfusion-fixed *in situ* using 4% buffered paraformaldehyde in PBS (4% PFA). After post-fixation in 4% PFA, hearts were embedded in Tissue-Tek O.C.T. compound, snap frozen, and cut into 5-µm transverse sections. Aortic root sections were collected when all three valves were clearly visible. Three sections 75 µm apart were stained by Aldehyde-fuchsin solution, and the average atherosclerotic plaque size was quantified. Quantification of lesion size in the aorta was assessed after staining lipid depositions using Oil-Red-O. The area of the aorta occupied by Oil-Red-O+ lipids was quantified by computerized image analysis (ImageJ 1.53, U. S. National Institutes of Health, Bethesda), expressed as the percentage of total aortic area. Necrotic core formation was quantified in sections stained with haematoxylin and eosin by measuring plaque area devoid of haematoxylin-stained cell nuclei. For immunofluorescence staining of macrophages and SMCs, sections were subjected to heat-induced antigen retrieval in citrate buffer. Sections were then blocked at room temperature (RT) for 30 min (PBS containing 2% mouse serum, 2% rabbit serum, 2% horse serum, 1% BSA, 0.1% Triton-X100). Antibody staining with rat anti-mouse Mac-2 (clone: M3/38, Biozol) and anti-mouse αSMA-Cy3 (clone: 1A4, Sigma Aldrich) was performed overnight at 4°C. After washing in PBS, incubation of the secondary antibody for Mac-2 was performed for 1h at RT using goat anti-rat Alexa Fluor 488 (Invitrogen). Sections were again washed in PBS and embedded in Vectashield mounting medium containing DAPI (Biozol) for nuclear staining. Images were captured with a Leica DM 4000B fluorescence microscope and JVC KY-F75U camera. Plaque size and cell content were quantified by computerized image analysis (ImageJ) and investigators blinded to the group distributions (2). The plaque vulnerability index was calculated by the ratio of monocyte/macrophage content (relative to the total plaque area) to the sum of SMC and collagen area (relative to the total plaque area).

Histological analysis
Liver, tail skin, colon and small intestine were removed and fixed in 4% PFA overnight before embedding in paraffin. Tissues were further sectioned at 1 or 5 µm, slides mounted and stained with haematoxylin and eosin (H&E). Sections were scored by experienced pathologists (M.B.-H., S.R-H. or A.R.) blinded to experimental history. Briefly, for the assessment of intestinal probes inflammation, crypt apoptotic body counts and crypt lost/destruction were semi-quantitatively scored and giant cells, ulceration/erosion and architectural distortion were evaluated as present or absent. From the respective values, a cumulative sum score was generated for each probe. For the analyses of skin probes dermal infiltrates were semi-quantitatively scored and acanthosis, spongiosis, hypergranulosis, lengthened/broadened rete ridges, basal vacuolization, subepidermal clefting, inflammatory cell exocytosis, apoptotic bodies and scab were evaluated as present or absent in 10 separate high-power fields and a sum score was generated from the mean values. For the assessment of liver tissue. For the assessment of liver tissue portal and lobular inflammation, stellate cell activation as well as liver cell regeneration were scored semi-quantitatively.

Serum cholesterol, triglycerides, and transaminases measurement
Serum total cholesterol (Amplex Red Cholesterol Assay Kit, Invitrogen) and triglycerides (EnzyChrom^{tm} Triglyceride Assay Kit, Bioassay Systems) were analyzed according to the

manufacturer's instructions and analyzed using an Infinite M200 PRO microplate reader (Tecan Life Sciences). Transaminases (AST and ALT) and total bilirubin were analyzed by an external laboratory with a Cobas 8000 automated system (Roche) with respective Roche kits for Cobas 8000 by photometric determination (Laboklin).

Serum lipid profile

Serum cholesterol lipoprotein profiles were determined by size exclusion chromatography (5). In brief, 3 µl of serum was fractioned using a Superose 6 3.2/300 gel filtration column from GE Healthcare (Uppsala, Sweden) and PBS, pH 7.4 as elution buffer, delivered by a first pump (Waters 1525 binary pump, Eschborn, Germany) at a flow rate of 50 µl/min. The separated lipoproteins were mixed in a T-tube with 50 µl/min cholesterol reagent (Roche, Mannheim, Germany) delivered by a second pump (Waters 1515 pump). Thereafter, the mixture went through a 500 µl reaction coil PEEK tubing (internal diameter 0.75mm) at 37°C in a post column reaction oven (Waters Temperature Control Module II). Finally, absorption was measured with an UV-VIS detector at 500 nm (Waters 2489 UV7Visible Detector). Total run time for each sample was 60 min. Chromatograms were integrated by Waters Empower 3 software. Very low-density lipoprotein (VLDL), LDL and high-density lipoprotein (HDL) concentrations were calculated as products of the area percent of total cholesterol.

Flow Cytometry

The aorta was carefully cleaned of fat and enzymatically digested, as described previously (2). Single-cell suspensions from blood, BM, spleen, aorta, inguinal and mesenteric lymph nodes (LN) were resuspended in PBS 1% FCS, and cells were stained with specific antibodies against CD3 (clone: 145-2C11, Thermo Fisher Scientific), CD4 (clone: RM4-5, Thermo Fisher Scientific), CD8α (clone: 53-6.7, Biolegend), CD11b (clone: M1/70, Biolegend), CD25 (clone: PC61, Biolegend), CD44 (clone: IM7, Thermo Fisher Scientific), CD45 (clone: 30-F11, Biolegend), CD62L (clone: MEL-14, Biolegend), Ly6C (clone: HK1.4, Thermo Fisher Scientific), CD115 (clone: AFS98, Thermo Fisher Scientific), TCRβ (clone: H57-597, Thermo Fisher Scientific) for 30 min at 4°C. Intracellular labelling of FoxP3 (clone: FJK-16s, Thermo Fisher Scientific), was performed according to the manufacturer's protocol (Thermo Fisher). Samples were acquired with FACSCelesta and analyzed with FlowJo 10.0 software (BD Biosciences).

Spectral flow cytometry

Single-cell suspensions of mouse aortas were incubated with normal rat serum (NRS) (1-part NRS to 20 parts PBS) for 5 minutes at 4°C to block unspecific binding to Fc receptors. Cells were stained with fluorochrome-labelled antibodies for 30 minutes at 4°C. Cells were co-stained with Zombie Aqua™ Kit (Biolegend) to exclude dead cells and with specific antibodies against CD3ε (clone: 145-2C11, Biolegend), CD4 (clone: GK1.5, Biolegend), CD8α (clone: 53-6.7, Biolegend), TCR Vg4-BUV395 (clone: 49.2-9, Biolegend), Ki67-BUV805 (clone: SolA15, Invitrogen), CD127-BV421 (clone: A7R34, Biolegend), CD178-SB600 (clone: MFL3, Invitrogen), CD184-BV711 (clone: L276F12, Biolegend), CD192-FITC (clone: SA203G11, Biolegend), CD44-PerCP/Cy5.5 (clone: IM7, Biolegend), CD195-PE (clone: C34-3448s, Biolegend), TNFα-PE-eFluor 610 (clone: MP6-XT22, Biolegend), Granzyme B-PE-Cy7 (clone: NGZB, Biolegend), TCF-7-AF647 (clone: 7F11A10, Biolegend) and CD122-APC-Vio770 (clone: TM-β1s, Biolegend). Samples were acquired on an Aurora spectral analyzer (Cytek Biosciences) using SpectroFlo Software (v.2.0) following daily quality control procedures as

instructed by the manufacturer. Acquired cytometry data were analyzed with FlowJo version 10 software (BD Biosciences).

Quantitative polymerase chain reaction (qPCR)

Total RNA was isolated from liver using Trizol reagent (Invitrogen). Prior to quantitative real-time PCR (qRT-PCR), liver RNA samples were reverse transcribed with the First Strand cDNA Synthesis Kit (Thermo Fisher Scientific) according to manufacturer's instructions. qPCR was performed on QuantiStudio 6 Flex Thermal Cycler (Applied Biosystems) using PowerUp™ SYBR™ Green Master Mix (Thermo Fisher Scientific). RPLP0 (Ribosomal Protein Lateral Stalk Subunit P0) was used as a housekeeping gene. The primers used in the study are listed in table 1.

scRNA-sequencing

Ldlr$^{-/-}$ mice underwent a BM transplantation and were fed a WD or normal chow as described above. Single cell RNA-seq was performed as described (6). After euthanasia, aortas from 4 mice per group were collected. Each aorta was individually digested, blocked, and stained with CITE-seq and hashtag antibodies before pooling for cytometry and fluorescence-activated cell sorting (FACS). Aortas were minced and digested for 90 minutes at 37°C under agitation in RPMI containing 450 U/ml collagenase I (Sigma- Aldrich, # C0130), 125 U/ml Collagenase XI (Sigma-Aldrich, C7657), 60 U/ml Hyaluronidase (Sigma- Aldrich, H3506), 60 U/ml DNAse (Roche # 11284932001), washed in PBS/1% FCS and passed through a 70 µm cell strainer to generate aortic cell suspensions. Cell suspensions were incubated for 5 minutes on ice with TruStain FcX™ (anti-mouse CD16/32, Biolegend # 101320, 10 µg/ml), and then labelled with anti-CD45.2-Alexa488 (clone 104, 2 µg/ml, Biolegend # 109816), Fixable Viability Dye e780 (Thermo Fisher # 65-0865-14, 1:1000) and anti-mouse TotalSeqA-Hashtag Antibodies 1 to 15 (1:250 dilution; all from BioLegend, TotalSeq™-A anti-mouse Hashtag antibodies) as follows: hashtag 1 to 4: BMT WD; hashtag 5-8: BM WD. (Hashtag 9-12 refer to samples not included in this study). After 25 minutes incubation at 4°C, cells were washed twice in PBS + 1% FCS and pooled. Viable CD45.2-APC (i.v.)^{neg} CD45.2-Alexa488^{+} were sorted using a FACS Aria III (BD Biosciences) with a 100µm nozzle. In this experiment, cells were labelled post-sort with a panel of TotalSeq-A CITE-seq antibodies against surface markers, but due to technical issues with the preparation of the CITE-seq cDNA library, the CITE-seq results are not interpretable. Cells were washed twice with PBS supplemented with 0.04% ultrapure BSA (Thermo Fisher AM2616), counted and loaded in the 10x Genomics Chromium with the aim to recover 10,000 cells.

Libraries were generated with the Chromium Single Cell 3' Reagents Kit v3. Samples were prepared for CITE-Seq/Hashing according to the manual until the cDNA amplification step in which 1 µl (0.2 µM) ADT PCR additive primer to capture Antibody-derived tags (ADTs) and 1 µl (0.1 µM) HTO PCR additive primer to capture Hashtag oligos (HTOs) were added. After cDNA amplification 60 µl (0.6x) SPRI beads (Beckman Coulter) were added to separate the supernatant fraction that contains the

ADT/HTO-derived cDNAs (< 180bp) and the bead fraction that contains the mRNA-derived cDNAs (> 300bp). The mRNA-derived cDNAs (bead fraction) were processed following the standard 10x Genomics protocol. The ADT/HTO-derived cDNAs (supernatant fraction) were purified twice with 2x SPRI. After the two purifications half amount of the eluted cDNAs were amplified for ADTs and half for hashtags. In the same reactions, the ADTs were indexed using TruSeq Small RNA primers and the hashtags using modified TruSeq DNA primers. The libraries were purified once more with 1.6x SPRI (160 µl). This method has been developed

by Stoeckius et al. and is described in detail in (7) and (8). Additionally, the detailed protocol including the oligo sequences can be accessed here: https://citeseq.com/protocol. All libraries were quantified by QubitTM 3.0 Fluometer (Thermo Fisher) and quality was checked using 2100 Bioanalyzer with High Sensitivity DNA kit (Agilent). Sequencing was performed with S1 or S2 100bp flow-cell with Novaseq 6000 platform (Illumina) and the reads for CITE-Seq/Hashing sample were allocated as follows: 5% for the hashtags, 10% for the ADTs and 85% for the mRNAs. 10x Genomics data, including HTO and ADT libraries, were demultiplexed using Cell Ranger software (version 3.1.0). Mouse GRCm38-mm10 reference genome was used for the alignment and counting steps. To evaluate the expression of the cell surface proteins alongside the transcriptome level in our data set, --feature-ref flag of Cell Ranger software was used which creates a matrix that contains gene expression counts alongside the expression of cell surface proteins.

Analysis of scRNA seq data

The gene-barcode matrix obtained from Cell Ranger was further analyzed using Seurat package from R (www.satijalab.org, version 4.1.1). First, data underwent quality control (mitochondrial gene expression), were normalized and scaled (linear transformed). Further downstream analysis, using only western diet fed *Ldlr*$^{-/-}$ mice, was performed using the top 2000 highly variable genes. A standard Seurat workflow was used to visualize the scRNA-seq data: Principal component analysis (PCA), Louvain Clustering (using a resolution parameter of 0.2) followed by Uniform Manifold Approximation and Projection (UMAP). Immune cell populations were identified using canonical marker genes. For re-clustering the lymphocyte containing cell population, the same workflow was performed as described above, using a clustering resolution parameter of 0.4. Different lymphocyte cell populations were identified using canonical marker genes. The statistical annotation feature ("stat.method") in the VlnPlot2 function built into the Seurat package was used to detect significantly differentially expressed genes, using a Wilcoxon rank-sum test with Bonferroni correction. Genes with adjusted $P<0.01$ were considered as differentially expressed genes. scRNA-Seq data has been uploaded to the Gene Expression Omnibus (GEO) database under accession number accession ID GSE291588.

Statistical analysis

Data are presented as mean ± standard deviation (std dev). Comparisons were performed via unpaired non-parametric Mann-Whitney U test with Prism software (GraphPad Prism 9). Differences in animal survival (Kaplan-Meier survival curves) were analysed by log-rank test. Differences were deemed statistically significant when $p < 0.05$.

Table 1

Primer	Sequence 5´ - 3´
mRplp0 - F	CCTATAAAAGGCACACGCGG
mRplp0 - R	CACGCGGGGTTTAAAGACG
mCyp7a1 - F	CACGCGGGGTTTAAAGACG
mCyp7a1 - F	GTGGACATATTTCCCCATCAGTT
Abcg5 - F	CTGCTGAGGCGAGTAACAAGAA
Abcg5 - R	GACGCATAATCACTGCCTGCT
Abcg8 - F	ACTTCAGGATGCTTCGCAGG
Abcg8 - R	TGCTCAAACCAAGGCACCTG
Abca1 - F	AGTGATAATCAAAGTCAAAGGGACAC
Abca1 - R	AGCAACTTGGCACTAGTAACTCTG
Hmgcr - F	CTTGTGGAATGCCTTGTGATTG
Hmgcr - R	AGCCGAAGCAGCACATGAT
Acat1 - F	CAGGAAGTAAGATGCCTGGAA
Acat1 - R	TTCACCCCCTTGGATGACATT
Cyp27a1 - F	TTTTGGCTGGGGTGGACA
Cyp27a1 - R	GGGCACCACACCAGTCACTT
Srebf2 - F	GACCTAGACCTCGCCAAAGGT
Srebf2 - R	AGCACGGATAAGCAGGTTTGTAG
Nr1h3- F	CCAGGAGATTGTGGACTTTGCC
Nr1h3- R	TCTGTCTCGTGGTTGTAGCGTC
Lrp1 - F	CGAGAGCCTTTGTGCTGGATGA
Lrp1 - R	CGGATGTCCTTCTCAATGAGGG
Vldlr - F	ACGGCAGCGATGAGGTCAACTG
Vldlr - R	CAGAGCCATCAACACAGTCTCG
Acat2- F	GAGATTGTGCCAGTGCTGGTGT
Acat2- R	GTGACAGTTCCTGTCCCATCAG
C6 - F	TGAGTGGCTAGAATCGGTGAAGG
C6 - R	GCTCTCCTGAGGTTGTTCCGTT
Folr2 - F	GTCACTTCATCCAAGACTCCTGC
Folr2 - R	CACTGGTGACAGTCCTCTTTGC
Grn - F	GTGTCTTGTGGTGATGGCTACC
Grn - R	GTCAGGACATTCAAACTGGCTCC
Il18bp - F	TCTCCAGCAGTCCCAACTAAGC
Il18bp - R	AGGCAGTACAGGACAAGGTCAG
Sdc3 - F	AGGAGCCTGATGTTGCTGAGAG
Sdc3 - R	CTCATCCTGGATGGTGGTCAGA
Themis - F	AGAAATCCAGTCAGGCATCTACTT
Themis - R	GACTCACAACCTCCAATAGCGC
Ifrd1 - F	CTGGCGAATCTTTGGCACTTCTG
Ifrd1 - R	ACCGCTGCTTTCTCTTGTCCAC
Nop58 - F	GGCTGGCATTTCCCTGAGTTAG
Nop58 - R	CTGCTTCTACTTCCTCAGACAGG

Socs2 - F	GCGCGTCTGGCGAAAGCCCT
Socs2 - R	GAAAGTTCCTTCTGGAGCCTCTT
Ccl2 - F	GCTACAAGAGGATCACCAGCAG
Ccl2 - R	GTCTGGACCCATTCCTTCTTGG
Il6 - F	TACCACTTCACAAGTCGGAGGC
Il6 - R	CTGCAAGTGCATCATCGTTGTTC
Il1b -F	TGGACCTTCCAGGATGAGGACA
Il1b-R	GTTCATCTCGGAGCCTGTAGTG
Tnf - F	GGTGCCTATGTCTCAGCCTCTT
Tnf- R	GCCATAGAACTGATGAGAGGGAG
Lipc-F	CTTCCAGCCTGGCTGCCACTT
Lipc-R	GCAAGGAGTCAATGAAGAGGTGC
Lpl-F	GCGTAGCAGGAAGTCTGACCAA
Lpl-R	AGCGTCATCAGGAGAAAGGCGA
Lipg-F	TACCTACACGCTGTCCTTTGGC
Lipg-R	GCTCGCATTTCACCATCTCTGAG

References

1. H. Shaikh *et al.*, Fibroblastic reticular cells mitigate acute GvHD via MHCII-dependent maintenance of regulatory T cells. *JCI Insight* **7** (2022).
2. J. Gil-Pulido *et al.*, Interleukin-23 receptor expressing gammadelta T cells locally promote early atherosclerotic lesion formation and plaque necrosis in mice. *Cardiovasc Res* **118**, 2932-2945 (2022).
3. E. Ullrich *et al.*, BATF-dependent IL-7RhiGM-CSF+ T cells control intestinal graft-versus-host disease. *J Clin Invest* **128**, 916-930 (2018).
4. J. G. Vargas *et al.*, A TNFR2-Specific TNF Fusion Protein With Improved In Vivo Activity. *Front Immunol* **13**, 888274 (2022).
5. J. Gil-Pulido *et al.*, Deletion of Batf3-dependent antigen-presenting cells does not affect atherosclerotic lesion formation in mice. *PLoS One* **12**, e0181947 (2017).
6. C. Cochain *et al.*, Single-Cell RNA-Seq Reveals the Transcriptional Landscape and Heterogeneity of Aortic Macrophages in Murine Atherosclerosis. *Circ Res* **122**, 1661-1674 (2018).
7. M. Stoeckius *et al.*, Simultaneous epitope and transcriptome measurement in single cells. *Nat Methods* **14**, 865-868 (2017).
8. M. Stoeckius *et al.*, Cell Hashing with barcoded antibodies enables multiplexing and doublet detection for single cell genomics. *Genome Biol* **19**, 224 (2018).

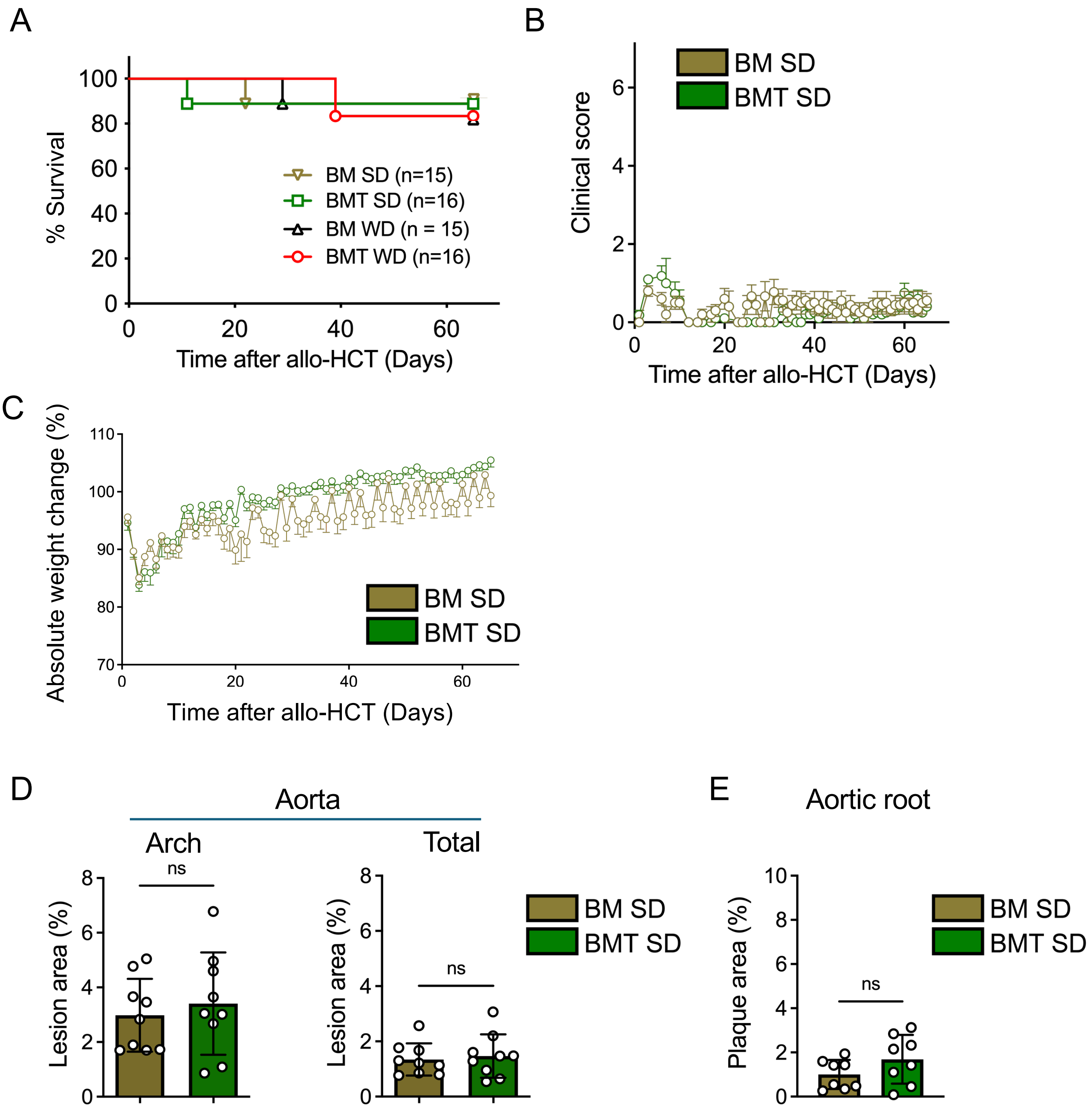

Figure S1. After myeloablative irradiation (9 Gy), miHAg-mismatched allogeneic 8-12 weeks old male B6.*Ldlr*$^{-/-}$ (H-2b) recipients were transplanted either with 5x10^{6} BM cells only or 5x10^{6} BM cells and 5x10^{6} enriched T cells (BMT group) from 8-12 weeks old BALB/b (H-2b) donors (BALB/b → B6.*Ldlr*$^{-/-}$). Starting on day 10 after HCT, B6.*Ldlr*$^{-/-}$ recipient mice were fed with western diet (WD) for 8 weeks, or fed a standard diet (SD). Combined data from two independent experiments are shown. **(A)** Kaplan-Meier survival curve. **(B)** Clinical GvHD score, **(C)** body weight change calculated as the percentage of the weight at the beginning of the experiment, and **(D)** quantification of lesion size in Oil-Red-O-stained aortas and **(E)** in Aldehyde-Fuchsin stained aortic roots in mice fed with SD. Data are presented as a mean ± std dev. Statistical significance was determined by unpaired non-parametric Mann-Whitney test. ns: non-significant.

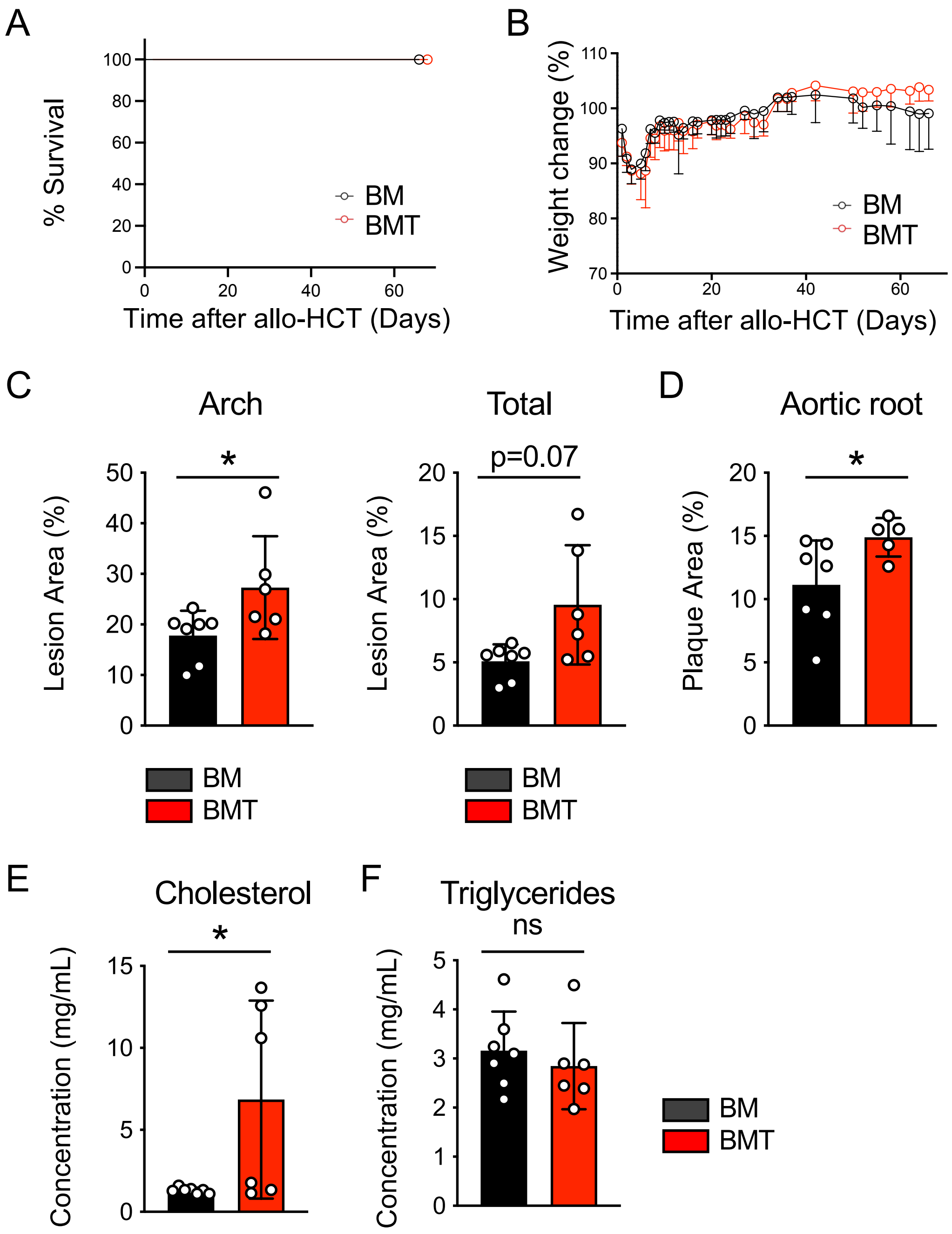

Figure S2. After myeloablative irradiation (9 Gy), female miHAg-mismatched allogeneic 8-12 weeks old B6.*Ldlr*$^{-/-}$ (H-2b) recipients were transplanted either with 5x10^{6} BM cells only (BM group, n=15) or 5x10^{6} BM cells and 5x10^{6} enriched T cells (BMT group, n=16) from 8-12 weeks old BALB/b (H-2b) donors (BALB/b → B6.*Ldlr*$^{-/-}$). Starting on day 10 after HCT, B6.*Ldlr*$^{-/-}$ recipient mice were fed with western diet (WD) for 8 weeks. **(A)** Survival and **(B)** body weight change calculated as the percentage of the weight at the beginning of the experiment. **(C)** Quantification of Oil-Red-O-stained aortas **(D)** and Aldehyde-Fuchsin stained aortic root sections. **(E, F)** Quantification of cholesterol and triglycerides in serum. Statistical significance was determined by unpaired non-parametric Mann-Whitney test. Data are presented as a mean ± std dev. *p < 0.05,, ns: non-significant.

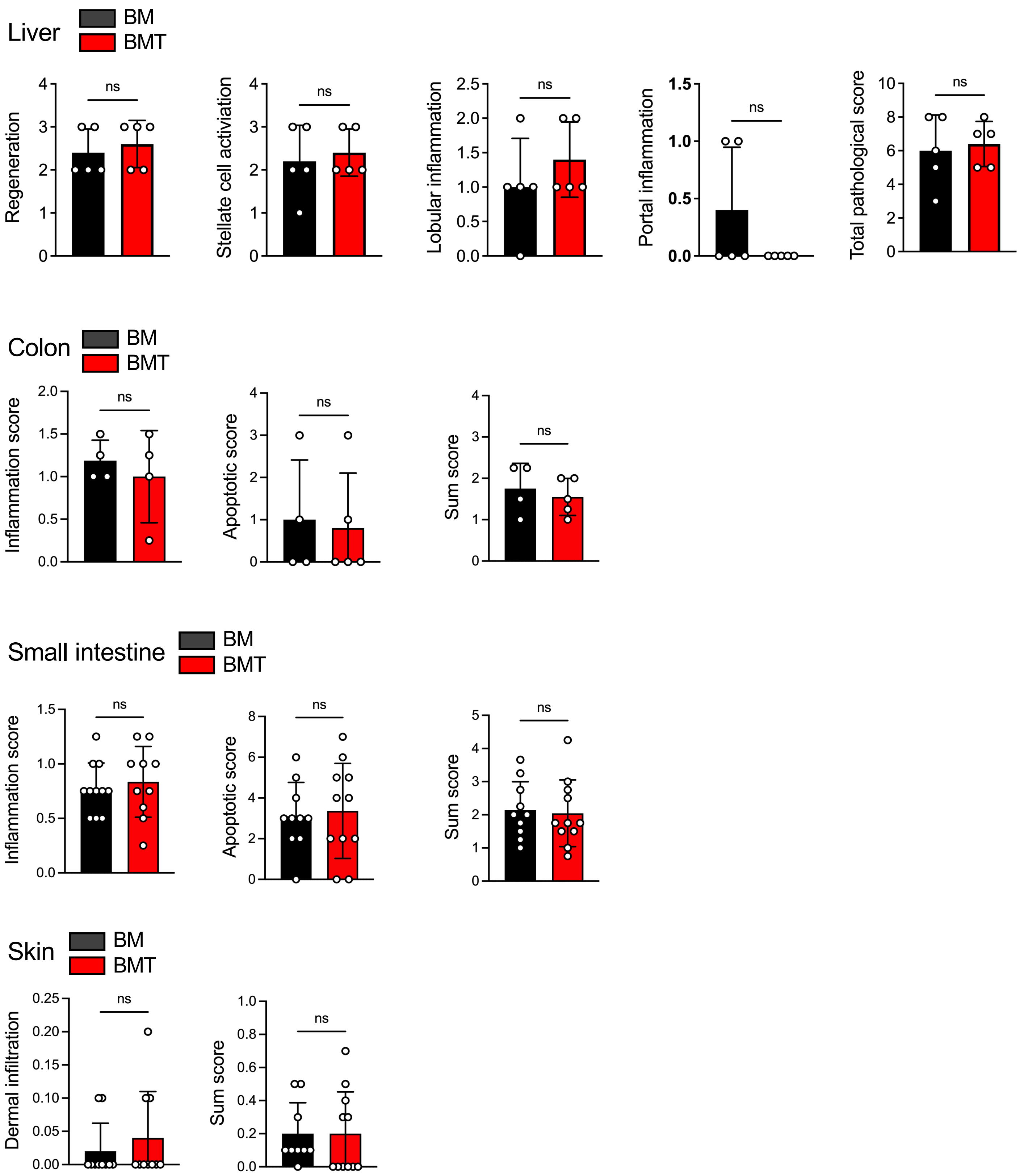

Figure S3. After myeloablative irradiation (9 Gy), male miHAg-mismatched allogeneic 8-12 weeks old B6.*Ldlr*$^{-/-}$ (H-2b) recipients were transplanted either with 5x10^{6} BM cells only (BM group, n=15) or 5x10^{6} BM cells and 5x10^{6} enriched T cells (BMT group, n=16) from 8-12 weeks old BALB/b (H-2b) donors (BALB/b → B6.*Ldlr*$^{-/-}$). Starting on day 10 after HCT, B6.*Ldlr*$^{-/-}$ recipient mice were fed with western diet (WD) for 8 weeks. Histopathological scoring of the liver, colon, small intestine and skin sections. Data are presented as a mean ± std dev, n=4-11. Statistical significance was determined by unpaired non-parametric Mann-Whitney test. ns: non-significant.

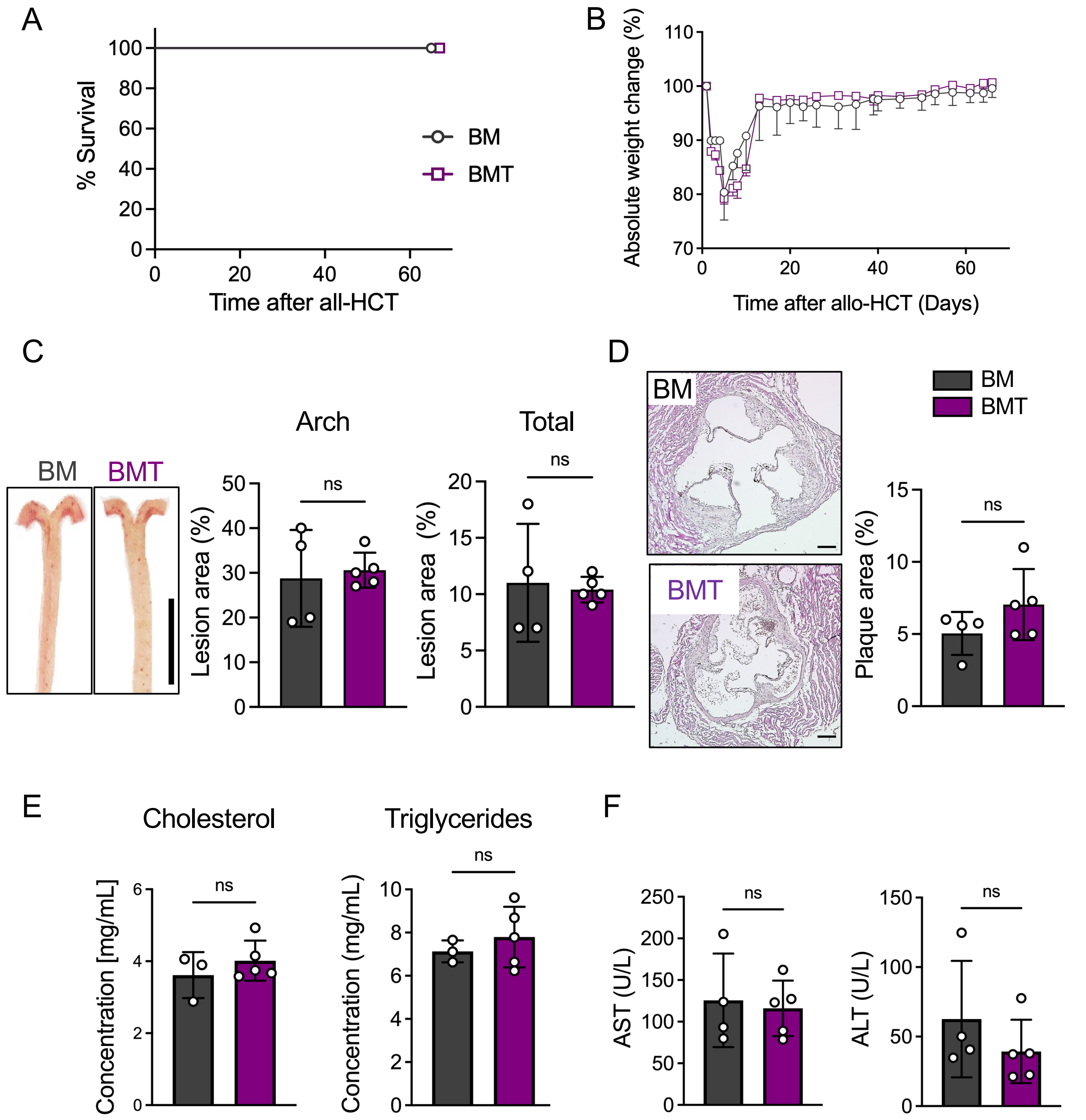

Figure S4. After myeloablative irradiation (9 Gy), syngeneic female B6.*Ldlr*$^{-/-}$ (H-2b) recipients were transplanted with 5x10^{6} BM cells (BM) or 5x10^{6} BM cells and 5x10^{6} T cells (BMT) from B6 (H-2b) donors (B6 → B6.*Ldlr*$^{-/-}$). Starting on day 10 after HCT, B6.*Ldlr*$^{-/-}$ recipient mice were fed a Western Diet for 8 weeks. **(A)** Kaplan-Meier survival curve. **(B)** Absolute weight change post allo-HCT. **(C)** Quantification of Oil-Red-O-stained aortas. Enface images of the aorta, scale bar, 1 cm. **(D)** Representative images and quantification of plaque area in H&E stained aortic root sections; scale bar, 100 µm. **(E)** Quantification of cholesterol and triglycerides levels in serum. **(F)** Quantification of AST and ALT in serum. Statistical significance was determined by unpaired non-parametric Mann-Whitney test. Data are presented as a mean ± std dev. ns: non-significant.

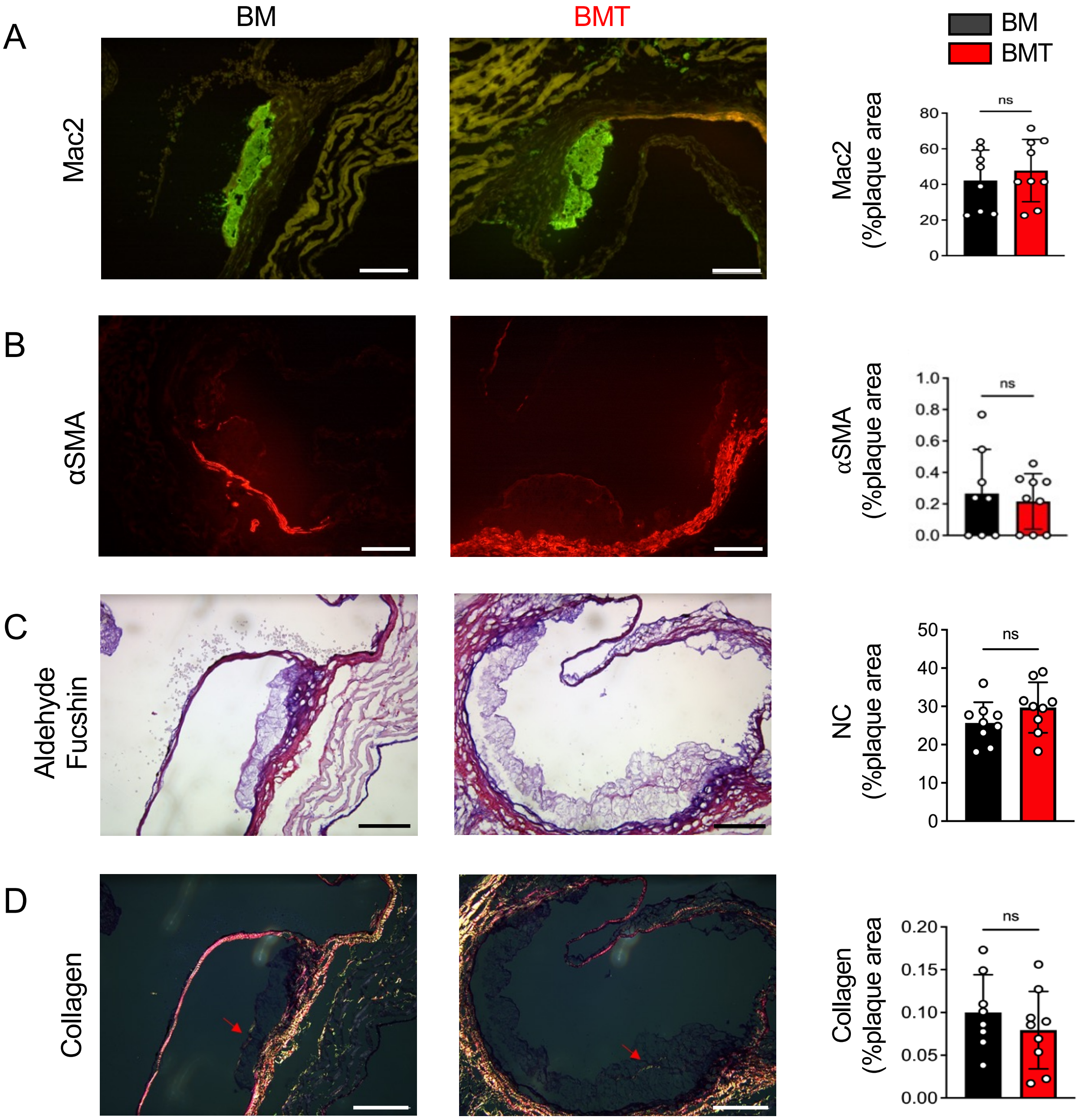

Figure S5. (A-D) After myeloablative irradiation (9 Gy), miHAg-mismatched allogeneic 8-12 weeks old maleB6.*Ldlr*$^{-/-}$ (H-2b) recipients were transplanted either with 5x10^{6} BM cells only (BM group, n=15) or 5x10^{6} BM cells and 5x10^{6} enriched T cells (BMT group, n=16) from 8-12 weeks old BALB/b (H-2b) donors (BALB/b → B6.*Ldlr*$^{-/-}$). Starting on day 10 after HCT, B6.*Ldlr*$^{-/-}$ recipient mice were fed with western diet (WD) for 8 weeks. Combined data from two independent experiments are shown, n=8-9. Quantification of the area positive for Mac-2 **(A)**, α-smooth muscle cell actin (αSMA) **(B),** necrotic core (NC) **(C)**, and collagen **(D)** relative to the total plaque area. Scale bars, 100µm. Data are presented as a mean ± std dev. Statistical significance was determined by unpaired non-parametric Mann-Whitney test. ns: non-significant.

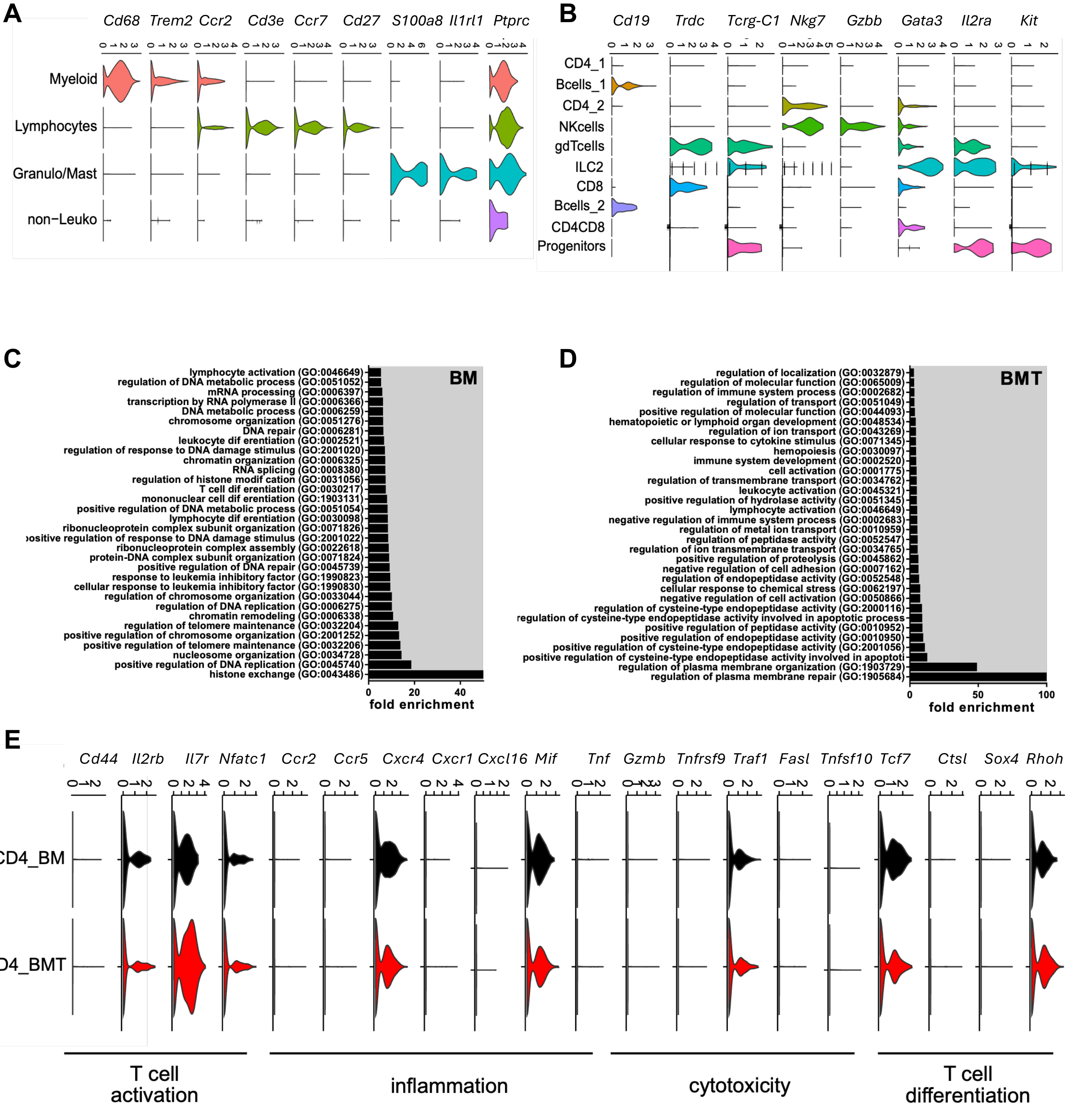

Figure S6. After myeloablative irradiation (9 Gy), miHAg-mismatched allogeneic 8-12 weeks old maleB6.*Ldlr*$^{-/-}$ (H-2b) recipients were transplanted either with 5x10^{6} BM cells only (BM group, n=15) or 5x10^{6} BM cells and 5x10^{6} enriched T cells (BMT group, n=16) from 8-12 weeks old BALB/b (H-2b) donors (BALB/b → B6.*Ldlr*$^{-/-}$). Starting on day 10 after HCT, B6.*Ldlr*$^{-/-}$ recipient mice were fed with western diet (WD) for 8 weeks. **(A)** Violin Plots of canonical marker genes to identify different leucocyte subsets. **(B)** Violin Plots showing gene expression for cluster characterization of different lymphocyte subclusters. Gene ontology analyses for enriched biological processes in BM group **(C)** and BMT group **(D)** showing only the top 32 biological processes. **(E)** Expression of genes in total CD4^{+} T cells indicative for T cell activation, inflammation, cytotoxicity and T cell differentiation comparing BM and BMT group.

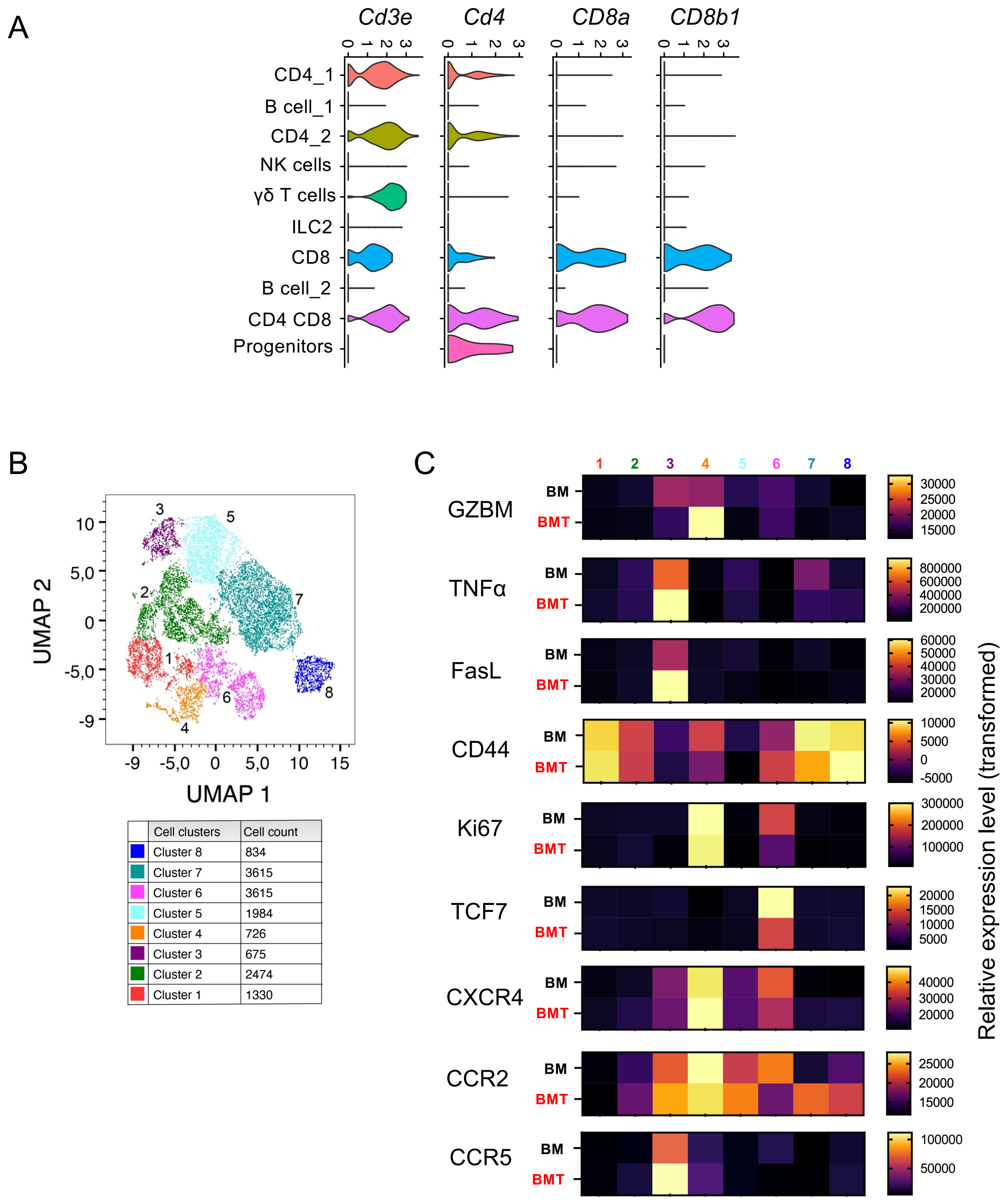

	Cell clusters	Cell count
	Cluster 8	834
	Cluster 7	3615
	Cluster 6	3615
	Cluster 5	1984
	Cluster 4	726
	Cluster 3	675
	Cluster 2	2474
	Cluster 1	1330

Figure S7. After myeloablative irradiation (9 Gy), miHAg-mismatched allogeneic 8-12 weeks old male B6.*Ldlr*$^{-/-}$ (H-2b) recipients were transplanted either with 5x10^{6} BM cells only (BM group, n=15) or 5x10^{6} BM cells and 5x10^{6} enriched T cells (BMT group, n=16) from 8-12 weeks old BALB/b (H-2b) donors (BALB/b → B6.*Ldlr*$^{-/-}$). Starting on day 10 after HCT, B6.*Ldlr*$^{-/-}$ recipient mice were fed with western diet (WD) for 8 weeks. **(A)** Violin Plots of canonical markers to identify CD4^{+} and CD8^{+} T cells in re-clustered lymphocytes after scRNA seq. **(B)** Cell clustering of 13094 cells into 8 translationally distinct cell cultures utilizing FlowSOM visualizing package on FlowJo. **(C)** Expression of Granzyme B, TNFα, Fasl, CD44, Ki67, TCF7, CXCR4, CCR2 and CCR5 on 8 cell clusters shown in heat-maps, quantification in relative expression level (transformed)

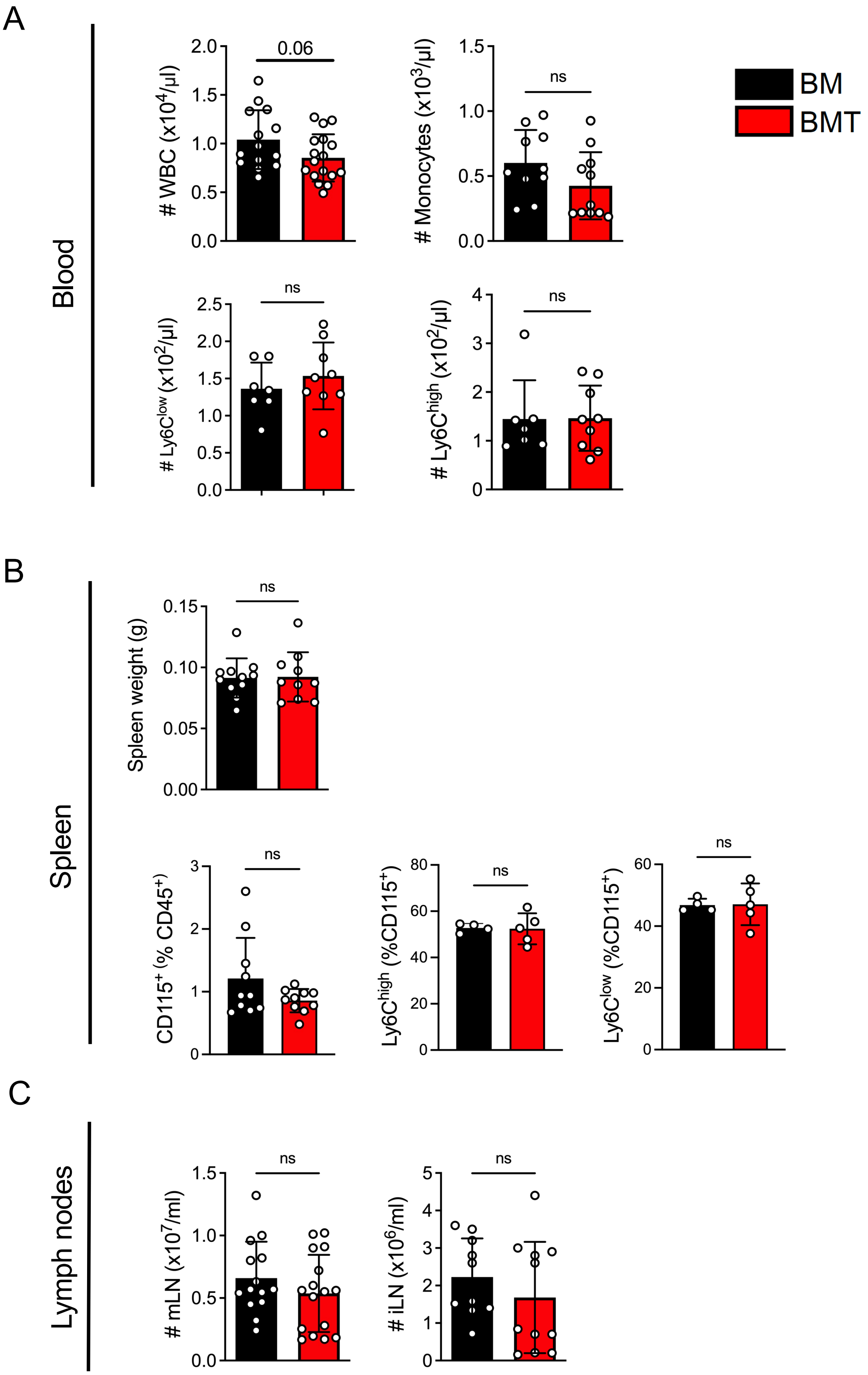

Figure S8. After myeloablative irradiation (9 Gy), miHAg-mismatched allogeneic 8-12 weeks old male B6.*Ldlr*$^{-/-}$ (H-2b) recipients were transplanted either with 5x10^{6} BM cells only (BM group, n=15) or 5x10^{6} BM cells and 5x10^{6} enriched T cells (BMT group, n=16) from 8-12 weeks old BALB/b (H-2b) donors (BALB/b → B6.*Ldlr*$^{-/-}$). Starting on day 10 after HCT, B6.*Ldlr*$^{-/-}$ recipient mice were fed with western diet (WD) for 8 weeks. Peripheral blood and spleen monocytes, lymph node cell number. Blood and spleen were analyzed by flow cytometry. **(A)** WBC number in blood, absolute number of monocytes, Ly6C^{high} and Ly6C^{low} in blood. **(B)** Spleen weight and relative number of monocytes, Ly6C^{high} and Ly6C^{low} in spleen are shown. **(C)** Cell number in mesenteric and inguinal lymph nodes. Data are presented as a mean ± std dev. Statistical significance was determined by unpaired non-parametric Mann-Whitney test. ns: non-significant.

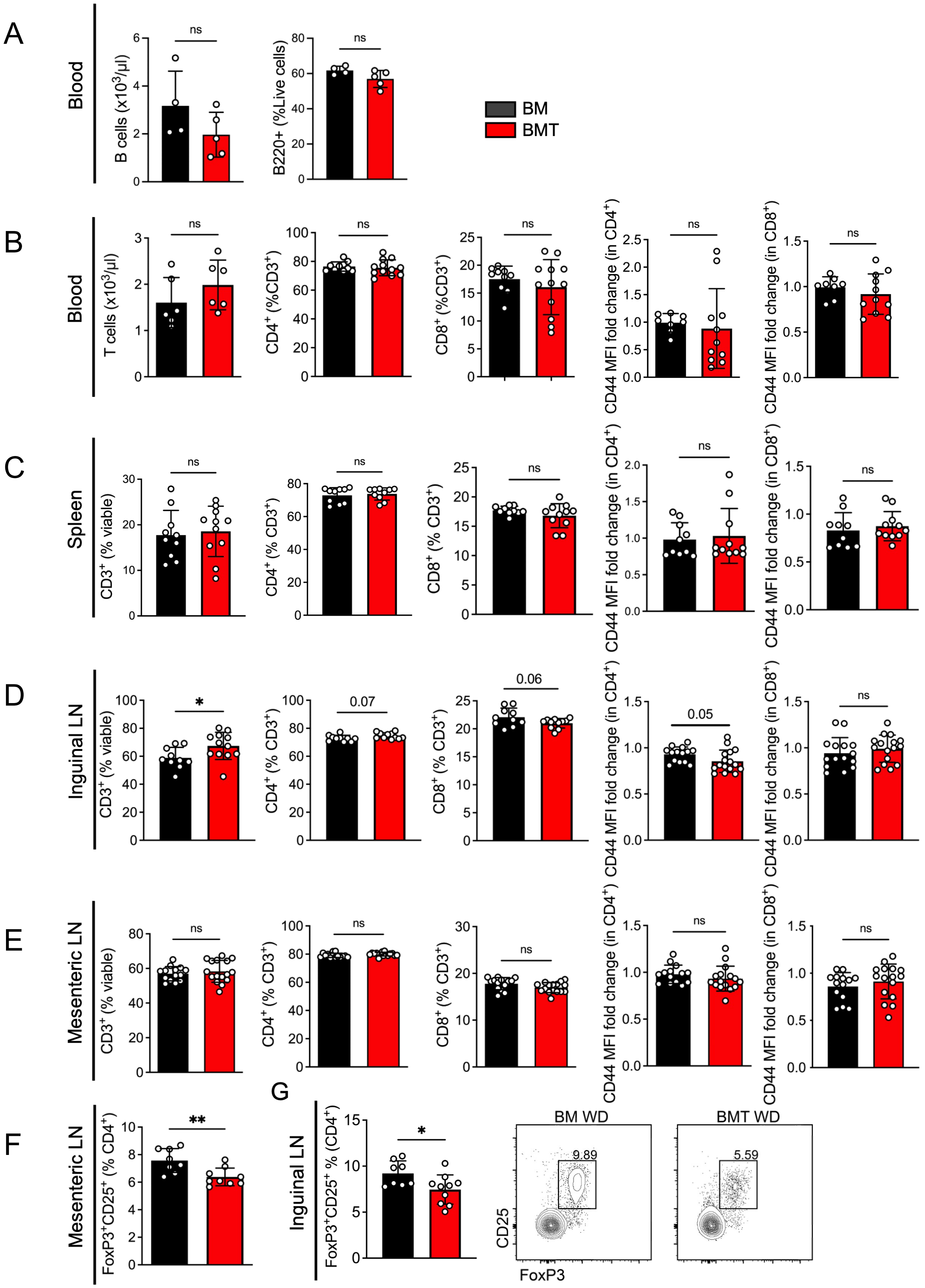

Figure S9. After myeloablative irradiation (9 Gy), miHAg-mismatched allogeneic 8-12 weeks old male B6.*Ldlr*$^{-/-}$ (H-2b) recipients were transplanted either with 5x10^{6} BM cells only (BM group, n=15) or 5x10^{6} BM cells and 5x10^{6} enriched T cells (BMT group, n=16) from 8-12 weeks old BALB/b (H-2b) donors (BALB/b → B6.*Ldlr*$^{-/-}$). Starting on day 10 after HCT, B6.*Ldlr*$^{-/-}$ recipient mice were fed with western diet (WD) for 8 weeks. Flow cytometry analysis was performed at day 66 post allo-HCT. Combined data from two independent experiments are shown, n = 4-11. **(A)** Quantification of B cells in peripheral blood and spleen. **(A-E)** T cells (CD3^{+}), CD8^{+} and CD4^{+} and expression of CD44 by CD4^{+} and CD8^{+} T cells in blood **(B)**, spleen **(C)**, and inguinal **(D)** and mesenteric LN **(E)**. Quantification of Tregs in mesenteric and inguinal LN with representative dot plot **(F)** is shown. Data are presented as a mean ± std dev. Statistical significance was determined by unpaired non-parametric Mann-Whitney test. *$p < 0.05$, **$p < 0.01$, ns: non-significant

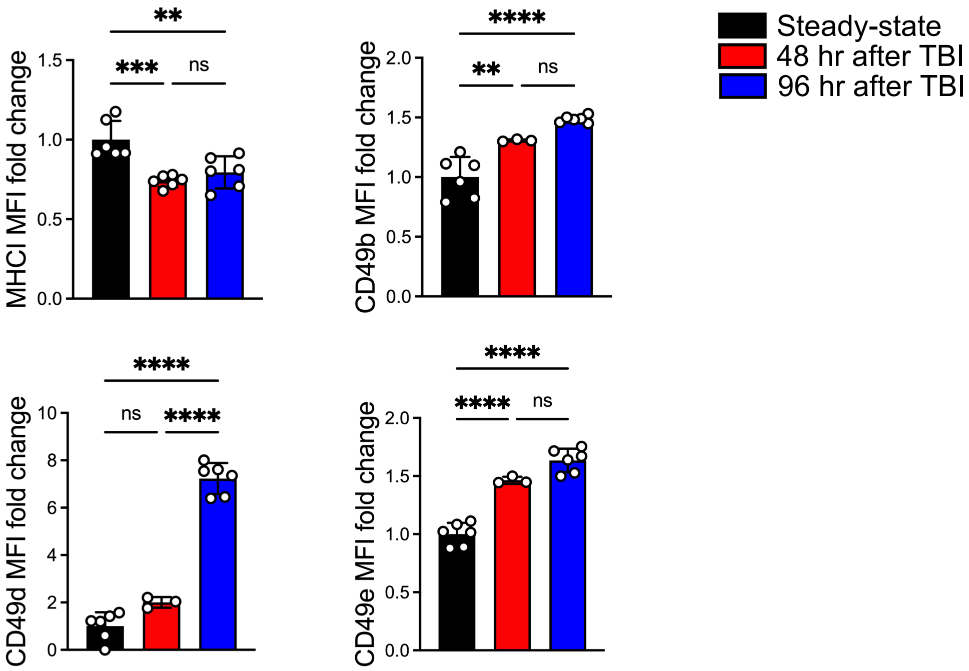

Figure S10. C57BL/6 mice were myeloablatively irradiated (9 Gy) or left untreated. Flow cytometry expression analysis of MHCI, CD49b (α2), CD49d (α4) and CD49e (α5) shown in histogram and quantification in MFI fold change was evaluated on heart endothelial cells ($CD45^{-}CD31^{+}$) at steady state (black), 48 hr after TBI (red), 96 hr after TBI (blue). Combined data from two independent experiments are shown. Statistical significance was determined by unpaired non-parametric Mann-Whitney test. Data are presented as a mean ± std dev. **$p < 0.01$, ***$p < 0.001$, ****$p < 0.0001$, ns: non-significant.

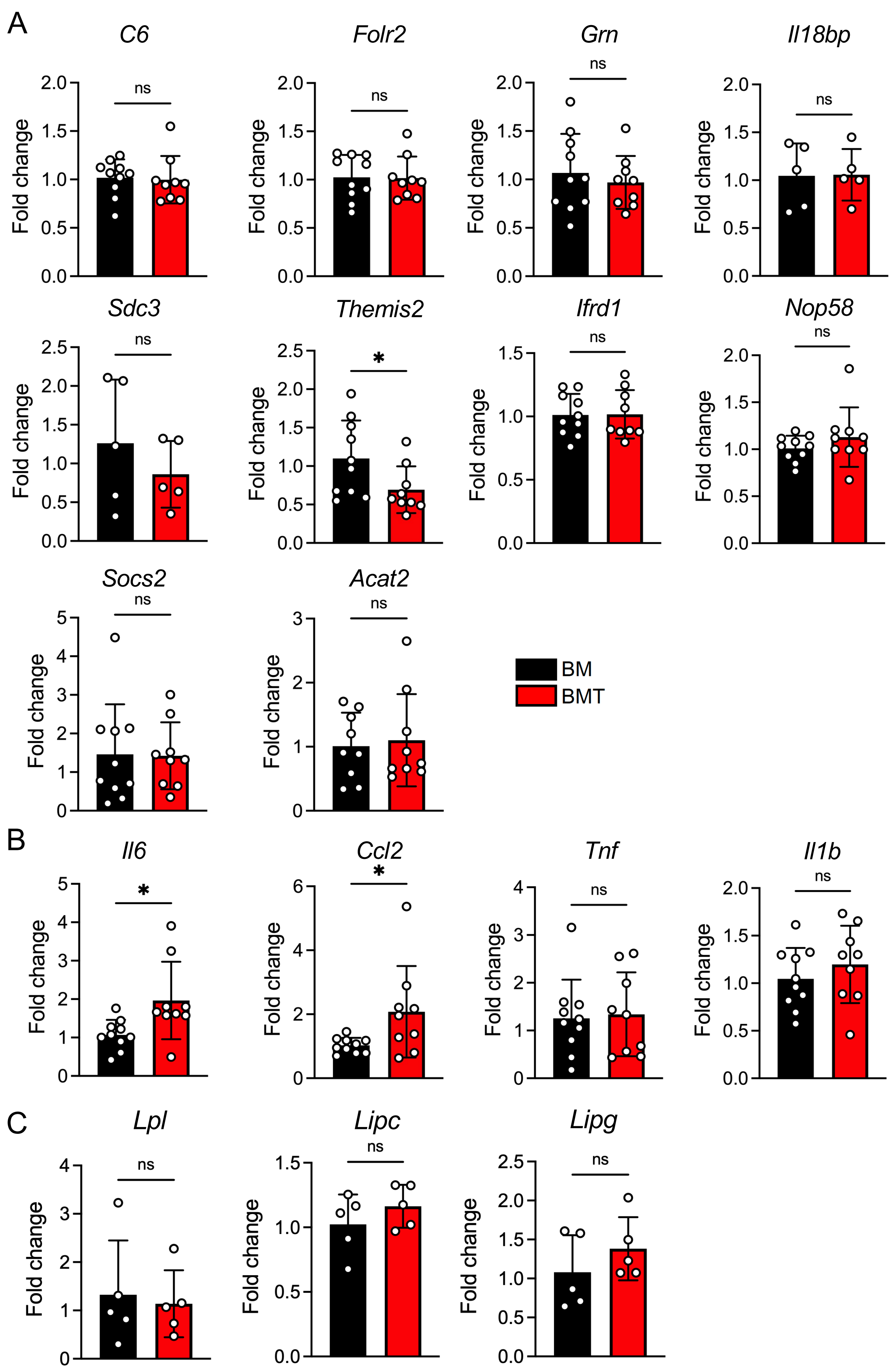

Figure S11. After myeloablative irradiation (9 Gy), miHAg-mismatched allogeneic 8-12 weeks old male B6.*Ldlr*$^{-/-}$ (H-2b) recipients were transplanted either with 5x10^{6} BM cells only (BM group, n=15) or 5x10^{6} BM cells and 5x10^{6} enriched T cells (BMT group, n=16) from 8-12 weeks old BALB/b (H-2b) donors (BALB/b → B6.*Ldlr*$^{-/-}$). Starting on day 10 after HCT, B6.*Ldlr*$^{-/-}$ recipient mice were fed with western diet (WD) for 8 weeks. **(A-C)** Liver inflammatory profiling relative mRNA fold change. **(A)** Expression of Kupffer cells related genes, **(B)** Expression of pro-inflammatory cytokines, **(C)** Expression of lipases. Data are presented as a mean ± std dev. Statistical significance was determined by unpaired non-parametric Mann-Whitney test. *p < 0.05, ns: non-significant.

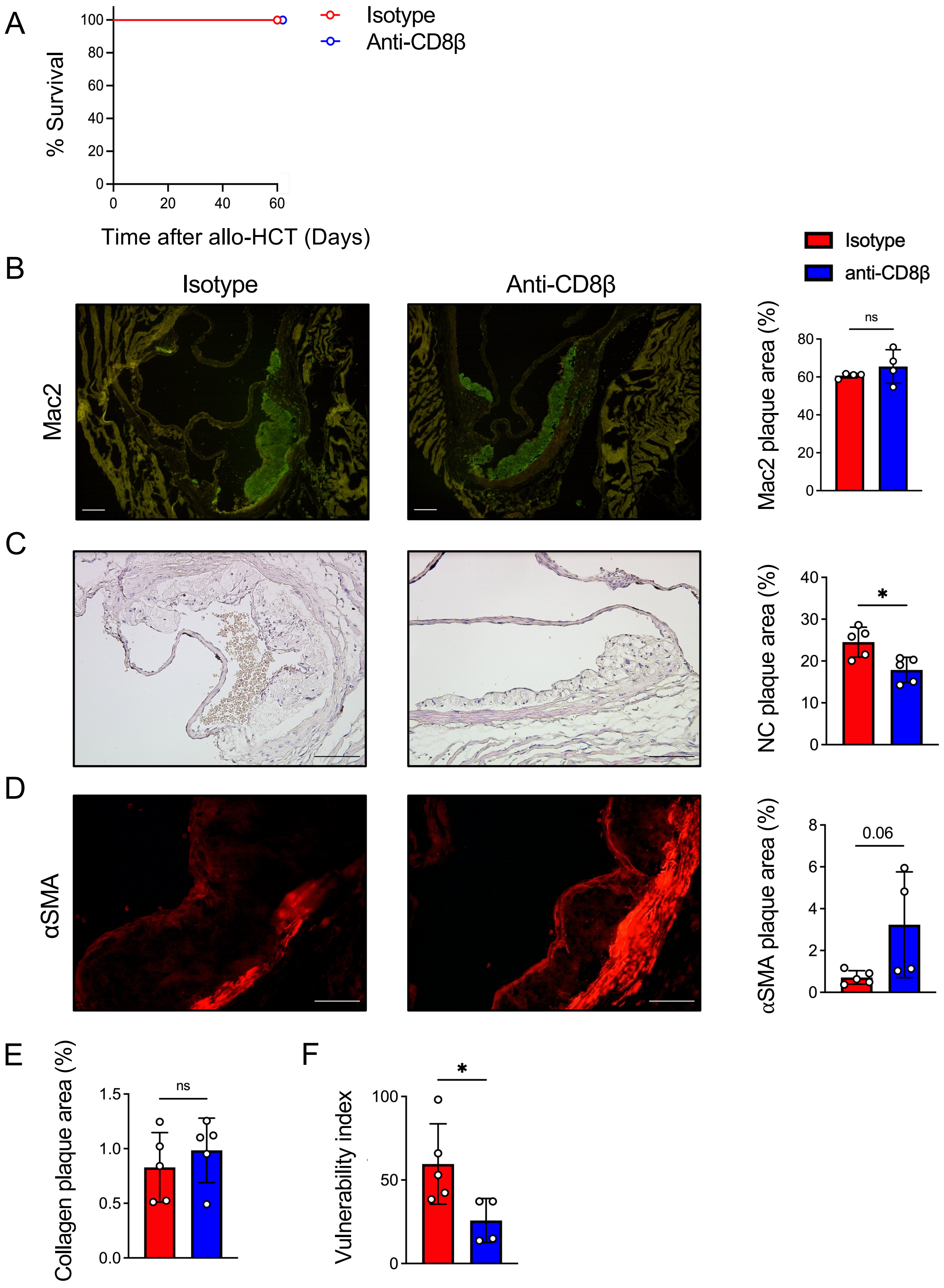

Figure S12. After myeloablative irradiation (9 Gy), miHAg-mismatched allogeneic 8-12 weeks old male B6.*Ldlr*$^{-/-}$ (H-2b) recipients were transplanted either with 5x10^6 BM cells only (BM group, n=15) or 5x10^6 BM cells and 5x10^6 enriched T cells (BMT group, n=16) from 8-12 weeks old BALB/b (H-2b) donors (BALB/b → B6.*Ldlr*$^{-/-}$). Starting on day 10 after HCT, B6.*Ldlr*$^{-/-}$ recipient mice were fed with western diet (WD) for 8 weeks, and injected (i.p.) with anti-CD8β antibody or isotype control antibody once per week (n=5 per group). **(A)** Kaplan-Meier survival curve. **(B)** Quantification of the area positive for Mac-2, **(C)** necrotic core, **(D)** α-smooth muscle cell actin, scale bar 50μm, presented as a percentage of a total plaque area, with corresponding images are shown. **(E)** Quantification of collagen in the plaque area and vulnerability index **(F)**. Data are presented as a mean ± std dev. Statistical significance was determined by unpaired non-parametric Mann-Whitney test. *p < 0.05, ns: non-significant.

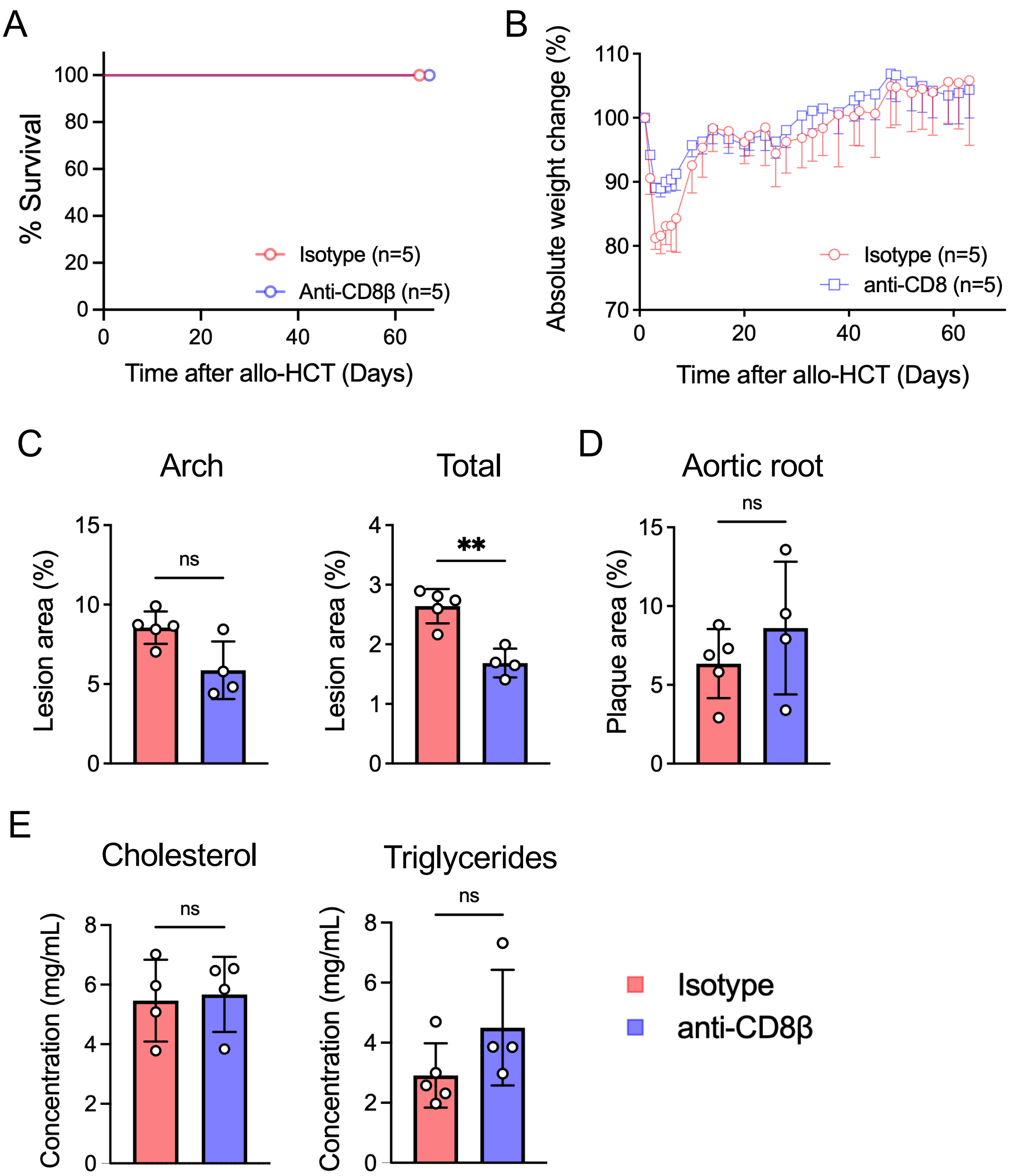

Figure S13. After myeloablative irradiation (9 Gy), miHAg-mismatched allogeneic 8-12 week old male B6.*Ldlr*$^{-/-}$ (H-2b) recipients were transplanted with 5x10^{6} BM cells only from 8-12 weeks old BALB/b (H-2b) donors (BALB/b → B6.*Ldlr*$^{-/-}$). Starting on day 10 after HCT, B6.*Ldlr*$^{-/-}$ recipient mice were fed with western diet (WD) for 8 weeks and injected (i.p.) with anti-CD8β antibody or isotype control antibody once per week (n=5 per group). **(A)** Kaplan-Meier survival curve. **(B)** Absolute weight change post BM transplantation. **(C)** Quantification of Oil-Red-O-stained aortas. **(D)** Quantification of plaque area in the aortic root. **(E)** Quantification of cholesterol and triglyceride levels in serum. Statistical significance was determined by unpaired non-parametric Mann-Whitney test. Data are presented as a mean ± std dev. **p < 0.01, ns: non-significant.
